# A Luminal Glycoprotein Drives Dose-Dependent Diameter Expansion of the *Drosophila melanogaster* Hindgut Tube

**DOI:** 10.1371/journal.pgen.1002850

**Published:** 2012-08-02

**Authors:** Zulfeqhar A. Syed, Anne-Laure Bougé, Sunitha Byri, Tina M. Chavoshi, Erika Tång, Hervé Bouhin, Iris F. van Dijk-Härd, Anne Uv

**Affiliations:** 1 Institute of Biomedicine, University of Gothenburg, Gothenburg, Sweden; 2 Centre des Sciences du Goût et de l'Alimentation, Université de Bourgogne, Dijon, France; Harvard Medical School, Howard Hughes Medical Institute, United States of America

## Abstract

An important step in epithelial organ development is size maturation of the organ lumen to attain correct dimensions. Here we show that the regulated expression of Tenectin (Tnc) is critical to shape the *Drosophila melanogaster* hindgut tube. Tnc is a secreted protein that fills the embryonic hindgut lumen during tube diameter expansion. Inside the lumen, Tnc contributes to detectable O-Glycans and forms a dense striated matrix. Loss of *tnc* causes a narrow hindgut tube, while Tnc over-expression drives tube dilation in a dose-dependent manner. Cellular analyses show that luminal accumulation of Tnc causes an increase in inner and outer tube diameter, and cell flattening within the tube wall, similar to the effects of a hydrostatic pressure in other systems. When Tnc expression is induced only in cells at one side of the tube wall, Tnc fills the lumen and equally affects all cells at the lumen perimeter, arguing that Tnc acts non-cell-autonomously. Moreover, when Tnc expression is directed to a segment of a tube, its luminal accumulation is restricted to this segment and affects the surrounding cells to promote a corresponding local diameter expansion. These findings suggest that deposition of Tnc into the lumen might contribute to expansion of the lumen volume, and thereby to stretching of the tube wall. Consistent with such an idea, ectopic expression of Tnc in different developing epithelial tubes is sufficient to cause dilation, while epidermal Tnc expression has no effect on morphology. Together, the results show that epithelial tube diameter can be modelled by regulating the levels and pattern of expression of a single luminal glycoprotein.

## Author Summary

Epithelial tubes constitute the functional units of vital organs, and they undergo highly regulated changes in size and shape during development to accommodate the three-dimensional configurations optimal for organ physiology. Through studies of *Drosophila melanogaster*, we show that epithelial tube diameter can be modelled simply by regulating the levels and pattern of expression of a single glycoprotein. The protein is secreted into the tubular lumen, where it forms a dense matrix and acts in a dose-dependent manner to drive diameter growth. We suggest that deposition of the protein into the lumen promotes local expansion of the lumen volume, and thereby stretching of the surrounding tube wall. Such a mechanism could represent a general means to adjust tube diameter during epithelial organ development.

## Introduction

Tube growth is a critical phase in the development of many organs and is tightly regulated to produce correct lumen dimensions. Tube-size maturation often occurs after the organ has acquired its basic layout and entails enlargement of the apical surface, and sometimes also expansion of the outer basal surface [1]. At the cellular level, tube growth is mediated by apical membrane growth, changes in cell shape and arrangement and cell proliferation. However, the signals that induce and steer these cellular changes to promote precise lumen size and shape are not fully understood.

It has become evident that lumen size can be influenced by the lumen environment itself. One example is through regulated osmotic pressure and fluid accumulation inside the lumen [2], [3], [4]. The resulting hydrostatic pressure will cause an increase in lumen volume and cellular remodelling within the surrounding epithelium to hold the larger lumen volume [5], [6], [7], [8]. A hydrostatic pressure can however not instruct a differential expansion of different regions of the same lumen, and requires that the epithelium has established a paracellular diffusion barrier [9], [10], [11]. Alternative means to shape organ lumens might involve luminal macromolecules. In the tracheal tubes of *Drosophila melanogaster*, lumen dilation depends on apical cell secretion [12], [13], but the formation of a uniform tube diameter and regular size of the apical cell domains requires a luminal chitin-based matrix that is transiently present during tube dilation [14], [15]. Moreover, lumen formation in the *Drosophila* retina requires Eyes Shut (Eys), a glycoprotein that is apically secreted by photoreceptor cells and causes separation of the apical membranes [16]. Similarly, the formation of a lumen during aortic tube formation in mouse requires de-adhesive functions of CD34-sialomucins that contribute to the apical glycocalyx and are thought to promote repulsion of the apical cell surfaces [17]. As the identity of luminal components in most developing organs has remained largely unknown, it is not clear to what extent they can contribute to the regulation of epithelial tube size.

In *Drosophila* embryos, it has been shown that mucin-type O-glycans are abundant in the lumen of many epithelial organs [18]. Mucin-type O-linked glycosylation is characterized by α-*N*-acetylgalactosamine (GalNAc) attached to the hydroxyl group of Serine and Threonine. In mucins, large domains containing repeats of Serine, Threonine and Proline (PTS-domains) become highly O-glycosylated and can form gel-like complexes upon binding to water due to the densely attached sugar residues [19], [20]. The *Drosophila* genome encodes several mucin-like proteins and, interestingly, a portion of these is dynamically expressed in embryonic epithelial organs [21], suggesting that they might be components of developing epithelial organ lumens with possible functions in tube growth. One such protein, Tenectin (Tnc), has indeed been shown to be secreted at the apical surface of the embryonic foregut, hindgut and tracheal tubes at mid-embryogenesis [22].

In this study, we explored a possible function for Tnc in epithelial tube growth, and found that Tnc is critical for diameter expansion of the hindgut. During hindgut growth, Tnc is observed as a dense striated matrix inside the lumen, and its luminal accumulation causes cell shape changes in the surrounding tube wall and tube expansion in a dose-dependent manner. Tnc exhibits limited spread along the tube axis, and can facilitate local dilation according to its spatial expression. The results suggest that Tnc drives volume expansion, and thereby tube dilation, and demonstrates a biological principle whereby the regulated expression of a single gene can steer the degree of lumen dilation along the tube length.

## Results

### Tenectin is an intraluminal protein in developing epithelial organs

**Figure 1:**
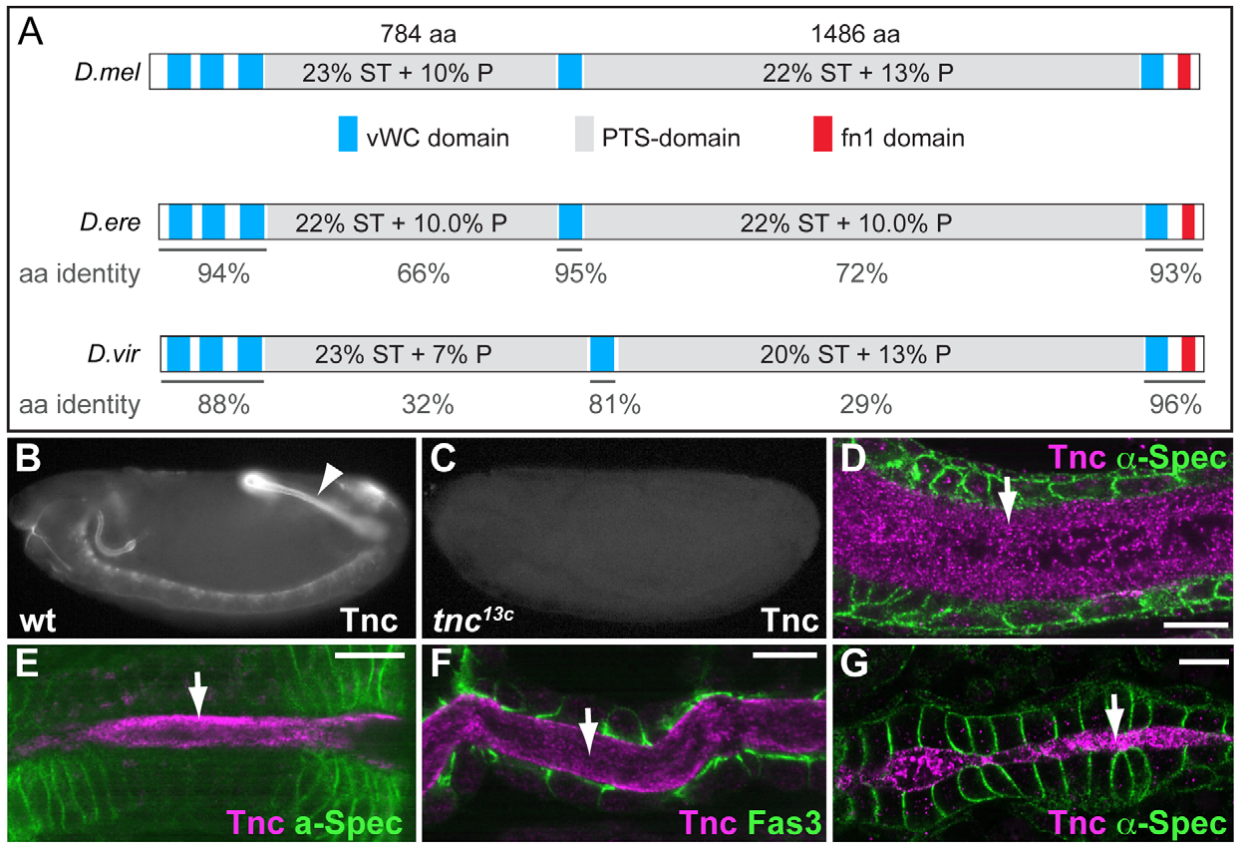
Tnc is a large protein detected in the lumen of developing epithelial tubes. (A) Comparison of Tnc from *D.melanogaster* (top) and predicted Tnc from *D.erecta* (middle) and *D.virilis* (bottom). The secreted proteins are drawn to scale, with the vWC-like domains in blue, PTS-domains in grey and the fibronectin-like domain in red. The PTS-domains of *D.melanogaster* Tnc show relatively low amino acid identity to those of *D.erecta* and *D.virilis* (represented as % below the domains), but their length and high P/T/S-content (indicated for the respective domains) are conserved between the species. (B and C) Anti-Tnc stains wild type embryos (B, arrowhead point to the hindgut), but not *tnc^13c^* mutant embryos (C). (D–G) Tnc (magenta) localizes to the lumen (arrow) of the hindgut (D, stage 15), foregut (E, stage 14), trachea (F, stage 15) and the dorsal vessel (G, stage 16). Epithelial cells are visualized by anti-α-Spec (green) in D, E and G, and by anti-Fas3 (green) in F. Scale bars: 5 µm. See also Figure S1.

Tnc is a protein of 2788 amino acids and is predicted to include an N-terminal signal peptide and no transmembrane domains. It harbours two extensive PTS-domains that are flanked by cysteine-rich domains with similarity to the von Willebrand factor type C (vWC) domain (Figure 1A). The PTS-domains are present also in predicted orthologs of Tnc in other *Drosophila* species (Figure 1A), but show poor amino acid identity between the species, although they are of similar lengths and are rich in Serine, Threonine and Proline (Figure 1A). The domain organization of Tnc therefore resembles that of secreted gel-forming mucins, in which large PTS-domains are separated by cysteine-rich von Willebrand factor-like domains that mediate polymer formation. In mucins, the sequences of PTS domains are not conserved between species, supporting that their major function is as a scaffold for O-linked carbohydrates [19].

We used anti-Tnc to detail the distribution of Tnc in embryonic epithelial organs. Tnc-staining was most prominent in the developing embryonic hindgut, (Figure 1B), where it localized to the hindgut lumen (Figure 1D). The *tnc* transcript is also present in the foregut at stage 14 and in the tracheal dorsal trunks at stage 15 [22]. Consistently, Tnc was detected in the lumen of the foregut (Figure 1E), proventriculus, salivary gland ducts (Figure S1) and the tracheal dorsal trunks (Figure 1F). In addition, we observed Tnc in the lumen of the dorsal vessel at late stage 16 (Figure 1G), which correlated with *tnc* mRNA expression in clusters of cardioblasts that form the dorsal vessel proper (Figure S1). In all organs analysed, Tnc distributed to the entire lumen and thus behaved as a secreted intraluminal component.

### Tnc is required for tube diameter-expansion of the hindgut

In order to address a possible function for Tnc in epithelial organ development, we generated *tnc* mutant alleles by imprecise excision of P-element EY16369 inserted between the two transcriptional start sites of *tnc* [23]. One of the excision alleles, *tnc^13c^*, carries a deletion of both *tnc* transcription start sites. No *tnc* mRNA (Figure S1) or Tnc protein (Figure 1C) could be detected in embryos homozygous for *tnc^13c^*, arguing that *tnc^13c^* is a loss of function allele.

**Figure 2:**
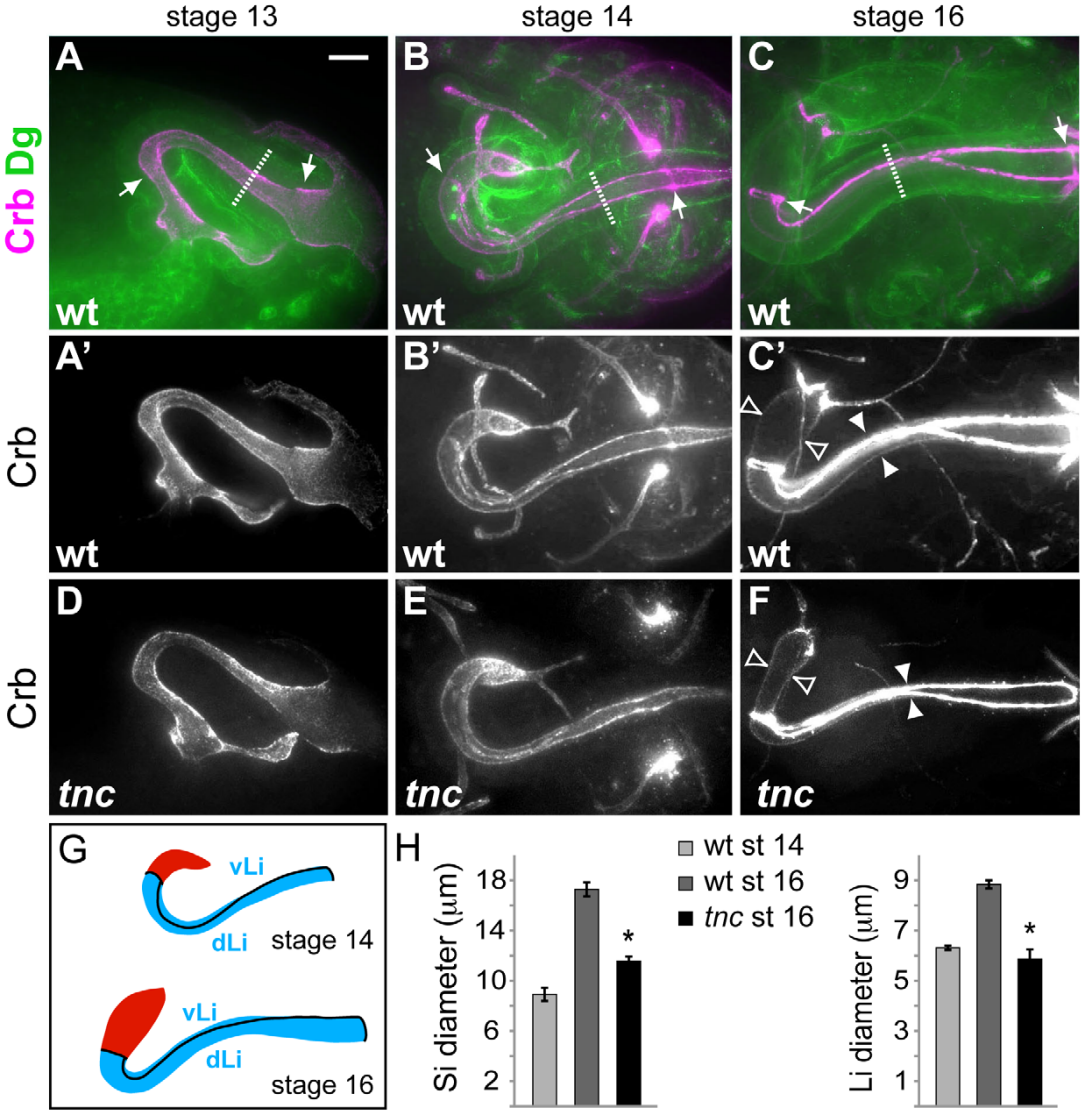
Tnc is required for hindgut lumen diameter expansion. (A–C) Wild type embryos were labelled for Crb (magenta) and Dg (green) to visualize the apical and basal surfaces of the hindgut epithelium. Arrows point to anterior and posterior Li borders, and stippled lines mark outer tube diameter. Crb-staining only is shown in A′–C′. At stage 13 (A, lateral view) the hindgut is narrow with an anterior hook pointing ventral. By stage 14, (B, dorsal view) the anterior hook has turned right and Crb-expressing border cells demarcate hindgut subdomains. From stage 14 to stage 16 (C, dorsal view) the hindgut expands in diameter and length. (D–F) *tnc^13c^* mutant embryos stained for Crb revealed a normal hindgut lumen at stage 13 (D), slight reduction in lumen diameter at stage 14 (E) and a clear reduction in lumen diameter at stage 16 (F), compared to the wild type. White and open arrowheads in (C′) and (F) illustrate lumen diameter of Li and Si, respectively. (G) Drawings of the hindgut lumen at stages 14 and 16 with Si in red and Li in blue. Border cells (black lines) mark anterior and posterior boundaries of Li and separates dorsal Li (dLi) and ventral Li (vLi). (H) The graphs show mean lumen diameter of Si and Li at stages 14 and 16 in the wild type and at stage 16 in *tnc^13c^* mutants. Li expands in diameter from 6.3 (+/−0.09) µm to 8.8 (+/−0.16) µm, and Si from 8.9 (+/−0.52) µm to 17.3 (+/−0.56) µm. * = P-value<0.05. Bars represent standard error of mean (n>5). Scale bar: 10 µm. See also Figures S2, S3, S4.

The shape of epithelial organ lumens of *tnc^13c^* mutant embryos was analysed by staining for Crumbs (Crb) that marks the apical epithelial surface. We found that *tnc^13c^* mutants had an unusually narrow hindgut lumen (compare Figure 2C′ and 2F). The same narrow hindgut was observed in embryos that carry *tnc^13c^* in trans to *Df(3R)BSC655*, a chromosomal deletion that lacks the *tnc* loci, and in embryos homozygous for *tnc^130a^*, an excision allele that lacks Tnc in the hindgut (Figure S2). Embryos homozygous for the viable *tnc^81b^* allele that corresponds to a precise excision of EY16369 have a hindgut similar to that of the wild type (Figure S2). It therefore appears that loss of *tnc* causes the narrow hindgut. We also noted that *tnc^13c^* mutant embryos exhibit slightly shorter tracheal dorsal trunks at stage 16, compared to the wild type (Figure S3), but could not detect any defects in other epithelial organs. Given the prominent expression and effect of Tnc in the hindgut, we focused on the function of Tnc in this organ.

The embryonic hindgut is an epithelial tube of ∼700 cells surrounded by a thin layer of visceral muscle cells [24], [25]. Hindgut formation commences with internalization of ectodermal cells at the posterior of the embryo [24], [25], [26]. The invagination elongates by mediolateral cell rearrangements to become narrow and J-shaped by stage 13 [24]. At this stage, the hindgut lumen of *tnc* mutants appeared similar to that of the wild type (compare Figure 2A′ and 2D).

By stage 14, the hindgut is divided into an anterior curved small intestine (Si), a posterior rectum and an in-between large intestine (Li). Li is further partitioned into a dorsal and ventral region, and all hindgut compartments are separated by Crb-expressing border-cells [27] (Figure 2G). Morphogenesis of the hindgut from stage 14 to 16 mainly involves tube elongation and expansion of tube diameter (Figure 2B′ and 2C′). Morphometric analyses showed that Li elongates by nearly 50% and Si almost triples in length from stage 14 to 16 (Figure S4). During the same period, Li and Si expand in diameter by about 40% and 95%, respectively (Figure 2H). The outer tube diameter, as visualized by anti-Dystroglycan (Dg), remained relatively constant during lumen growth (Figure 2A–2C), which is consistent with a concurrent flattening of the tubular epithelium [24]. In *tnc* mutant embryos, the hindgut lumen appeared slightly narrow at stage 14, when compared to the wild type (Figure 2B′ and 2E), and the difference in diameter increased until stage 16 (Figure 2C′ and 2F). The narrow lumen was not a result of the fixation, since it was also evident when analysing live embryos (Figure S2). At stage 16, Li diameter was similar to, and Si diameter was only 25% wider than that of the wild type at stage 14 (Figure 2H). The length of Li and Si lumens was however not reduced in *tnc* mutants, and Li was slightly longer in the mutants relative to the wild type (Figure S4). Assuming a circular lumen circumference, the hindgut lumen volume of *tnc* mutants would be less than 50% of that in the wild type at stage 16. Tnc is therefore critical for diameter expansion of the hindgut, after establishment of the basic organ layout at mid-embryogenesis.

### Tnc-mediated lumen dilation depends on cell shape changes

**Figure 3:**
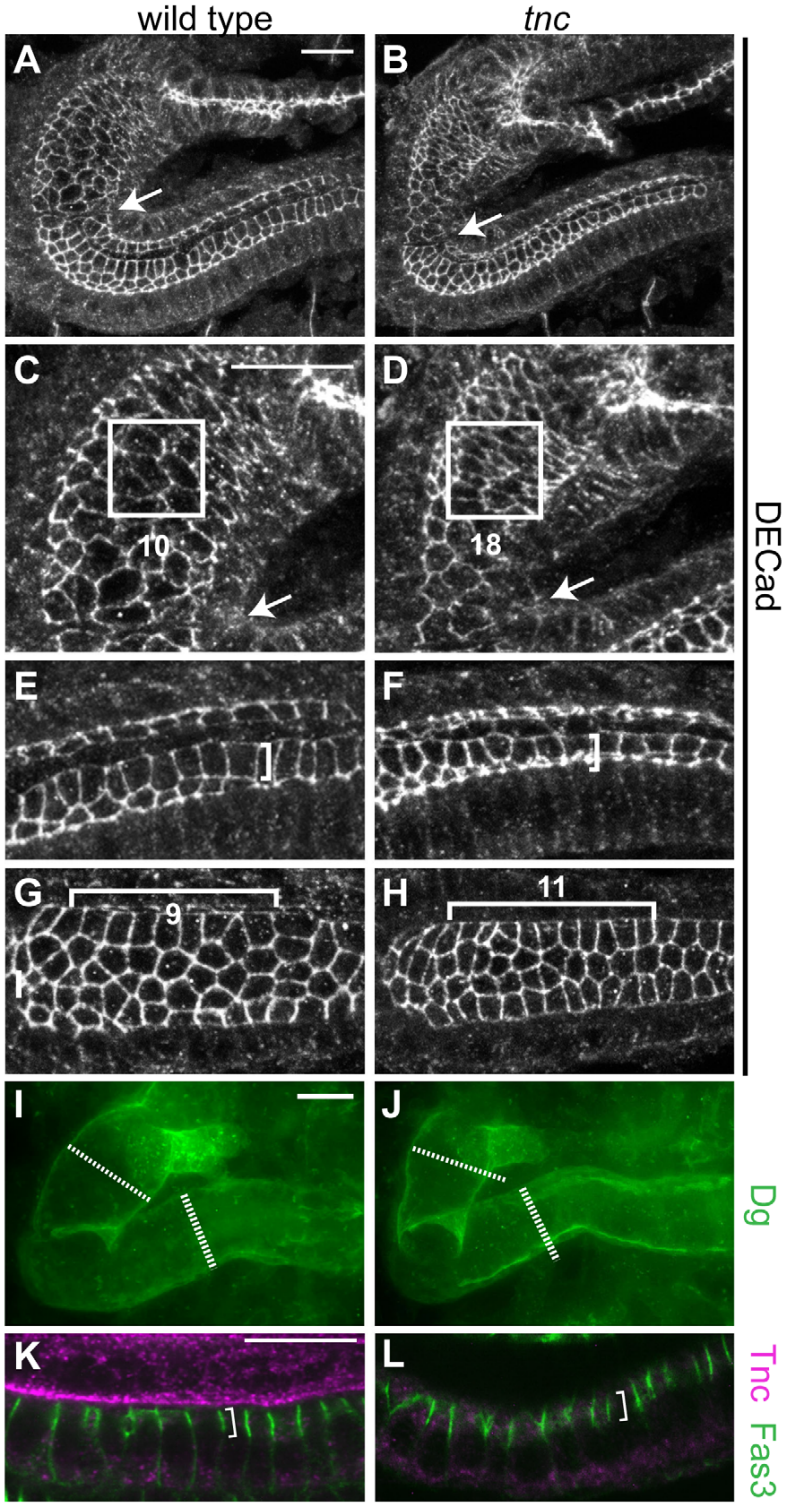
Loss of *tnc* causes reduced apical cell circumferences, altered cell arrangement, and a smaller outer tube diameter. (A–H) Embryos were labelled with anti-DECad, and serial z-stacked images spanning the upper half of the hindgut tube were merged to reveal apical cell circumferences. Arrows point to the Si/Li border. The hindgut of a wild type and *tnc* mutant embryo at dorsal view (A and B) is shown together with high magnifications of respective Si (in C and D) and posterior Li (in E and F). Identically sized squares (in C and D) span ∼10 cells of the wild type Si and ∼18 cells of the mutant Si. Brackets of identical size (in E and F) illustrate that cells are more elongated along the lumen perimeter in the wild type compared to the mutant. In the dorsal Li (G and H, ventral view), identically sized brackets span 9 or 11 cells along the border cells in the wild type and mutant hindgut, respectively. The mutant hindgut also has fewer cells at the dorsal lumen perimeter. (I and J) The outer hindgut diameter, visualized by labelling for Dg (green) and merging serial z-stacked images that span the entire hindgut, is reduced in the mutants. Stippled and full lines represent wild type Si and Li diameter, respectively. (K and L) Labelling with anti-Fas3 (green) reveal comparable level and distribution of Fas3 (bracket) in the hindgut epithelium of wild type (K) and *tnc* mutant (L) embryos. Co-staining for Tnc (magenta) shows the absence of Tnc in the mutant hindgut. All images are of stage 16 embryos. Scale bars: 10 µm in A (A and B), 10 µm in C (C–H), 10 µm in I (I and J), 10 µm in K (K and L).

Growth of the wild type hindgut occurs, from stage 13, through increase in cell size, change in cell shape and cell rearrangement [24]. To address the cellular changes that are associated with the narrow hindgut in *tnc* mutants, we stained for Drosophila epithelial cadherin (DECad) to reveal the apical cell circumferences. By counting cells within different segments of the hindgut epithelium, we could not detect differences in cell number (data not shown), but the cells of the mutant hindgut showed reduced apical circumferences (Figure 3A and 3B). The smaller apical cell domains were particularly evident in Si, where the number of cells covering identical sized areas was 1.8 times higher in *tnc* mutants than in the wild type (Figure 3C and 3D).

A close examination of the apical cell circumference in Li also revealed small aberrations in cell arrangement in *tnc* mutants, which were consistent with the narrow tube diameter: In the posterior Li, cells were less stretched along the lumen circumference (illustrated by identically sized brackets in Figure 3E and 3F), although the cells appear equally stretched along the tube length. In the anterior Li, there were more cells along the lumen length (11 versus 9 cells over 16 µm) and fewer cells surrounding the lumen circumference (Figure 3G and 3H), indicating a higher degree of cell intercalation in the this part of the Li.

The use of anti-Dg to visualize the basal hindgut surface revealed that also the outer tube diameter was consistently reduced in stage 16 *tnc* mutants. Serial z-stacked images spanning the entire hindgut were merged to yield a representative outer diameter (Figure 3I and 3J). Together, these observations argue that Tnc is required to expand the entire tube wall, by a mechanism associated with cell shape changes and slight cell rearrangements, similar to the effects caused by a luminal osmotic pressure [8].

In the above studies, we did not detect differences in the level or localization of Crb and DECad between the wild type and mutant hindgut epithelia. The mutant hindgut also showed normal level and localization of Fasciclin 3 (Fas3), a marker for septate junctions that is present along the apico-lateral cell surface (Figure 3K and 3L). Finally, staining for the transcription factor, Myocyte enhancer factor 2, revealed normal presence of visceral muscle cells surrounding the mutant hindgut epithelium, and expression of Delta and Engrailed in the ventral and dorsal halves of Li was unaffected in the mutants (data not shown). Tnc had therefore no detectable effects on the patterning or epithelial integrity of the hindgut tube.

### Tnc drives dilation of the hindgut in a dose-dependent manner

**Figure 4:**
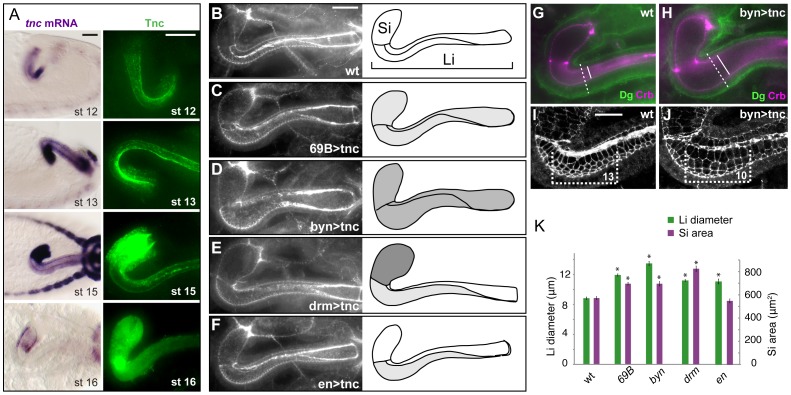
Tnc promotes hindgut diameter-expansion in a dose-dependent manner. (A) The distribution of *tnc* transcripts (left) and Tnc protein (right) is shown in the hindgut for stages 12 to 16. At stage 12 (lateral view) *tnc* expression is prominent in the ventral hindgut, and anti-Tnc densely stains the ventral lumen at stages 12 and 13. From stage 13 to 15 (dorsal views), *tnc* expression is higher in Si than in Li, and Tnc is seen in intracellular puncta in Si. At stage 16 (dorsal views), *tnc* mRNA levels decline and Tnc is detected only inside the lumen. (B–F) Embryos were labelled for Crb and DECad (both in white) to visualize hindgut lumen size in the wild type (B) and in embryos that express *UAS*-*tnc* in the hindgut driven by *69B-GAL4* (C), *bynGAL4* (D), *drmGAL4* (E) and *enGAL4* (F). All images are projections of serial z-stacks at dorsal view. Drawings of the respective lumens (right) also indicate the pattern of GAL4 expression (dark shading represents strong GAL4 expression). The size of lumen diameter correlated with GAL4 expression level. (G–J) Wild type and *UAS-tnc/BynGAL4* (byn>tnc) embryos were co-labelled with anti-Crb (magenta) and anti-Dg (green) (G and H) and with anti-Crb and anti-DECad (both in white, I and J). Compared to the wild type, byn>tnc embryos have enlarged outer and inner tube diameter (stippled and full lines in G and H) and a flattened tubular epithelium (compare difference in inner and outer diameter in G and H). byn>tnc embryos also show enlarged apical cell circumferences and fewer cells along the Li tube axis (I and J, equal-sized brackets span 13, respective 10, cells along the dorsal-ventral boundary). (K) The mean diameter of Li (green) and area of Si (magenta) are shown for wild type embryos and embryos with GAL4-driven *tnc* expression. Note that *drmGAL4*, which drives strong expression in Si, causes a relatively larger dilation of Si than Li when compared to the other genotypes. P-value<0.05. Error bars represent standard error of mean (n = 8). Scale bars: 20 µm in A, 20 µm in B (B–F), 10 µm in I (I and J). See also Figures S5 and S6.

Our analyses show that Tnc is required for expansion of the hindgut during stages 14 to 16, and that loss of Tnc resulted in a more severe reduction in Si diameter (6 µm) than in Li diameter (3 µm). To understand how these observations correlate with the presence of Tnc in the hindgut lumen, we followed Tnc expression during hindgut development (Figure 4A). From stage 13 to 16, Tnc appeared to gradually accumulate inside the lumen, consistent with a robust expression of *tnc* mRNA in the hindgut epithelium during stages 13 to 15. Transcript levels were highest in Si, and detection of Tnc as intracellular puncta in Si presumably represents Tnc protein under secretion. By stage 16, Tnc was abundant in the hindgut lumen. However, Tnc was not detected inside the cells and *tnc* mRNA expression had declined, indicating a cessation of Tnc synthesis and secretion into the lumen.

The stronger expression of *tnc* in Si versus Li, suggested that the degree of diameter expansion might correlate with the levels of *tnc* expression. To address this possibility we analysed the effect of Tnc over-expression in the hindgut. The P-element *EY16369*, inserted between the two transcriptional start sites of *tnc*, contains binding-sites for the GAL4 transcription factor and promotes transcription of *tnc* in the presence of GAL4 [23]. We first over-expressed Tnc uniformly in the hindgut epithelium using *69B-GAL4*, which drives ubiquitous expression in the ectoderm from late stage 9 [28], [29], and *bynGAL4*, which drives strong expression in the hindgut starting at stage 7 [30] (Figure S5). Tnc over-expression resulted in an excessively dilated hindgut lumen at stage 16 (Figure 4B–4D). The effect was strongest with *bynGAL4*, yielding a Li diameter 1.5 times that of the wild type, while over-expression with *69B-GAL4* caused a 1.4 increase in Li diameter (Figure 4K). Si exhibited a ballooned appearance upon over-expression of Tnc, and the size of the Si lumen is therefore presented as an area. We found that both *bynGAL4-* and *69B-GAL4*-driven *tnc*-expression caused a 1.2 times increase in Si area (Figure 4K). The excessively dilated lumen was accompanied by an increase also in outer tube diameter and by flattening of the tube wall (Figure 4G and 4H), and labelling with DECad showed enlarged apical cell circumferences in the hindgut and fewer cells along the tube, when compared to the wild type situation (Figure 4I and 4J). These effects are opposite to those observed in *tnc* mutant embryos. There was no significant alteration in hindgut length in embryos that over-express Tnc (Figure S6). It therefore appears that Tnc is sufficient to drive expansion of the hindgut lumen, and that it does so in a dose-dependent manner.

The GAL4-line, *drmGAL4*, drives expression of UAS-transgenes in the hindgut epithelium [31], but at a higher level in Si compared to Li (Figure S5). We used *drmGAL4* to test if the pattern of *tnc* expression along the hindgut tube would be reflected by a differential degree in tube dilation. Indeed, expression of Tnc driven by *drmGAL4* resulted in a 1.44 times Si area and a moderate 1.27 times Li diameter when compared to the wild type (Figure 4E and 4K). Thus, Tnc can cause local tube dilation according to its pattern and levels of expression.

### Tnc is an O-glycosylated matrix component of the hindgut lumen

**Figure 5:**
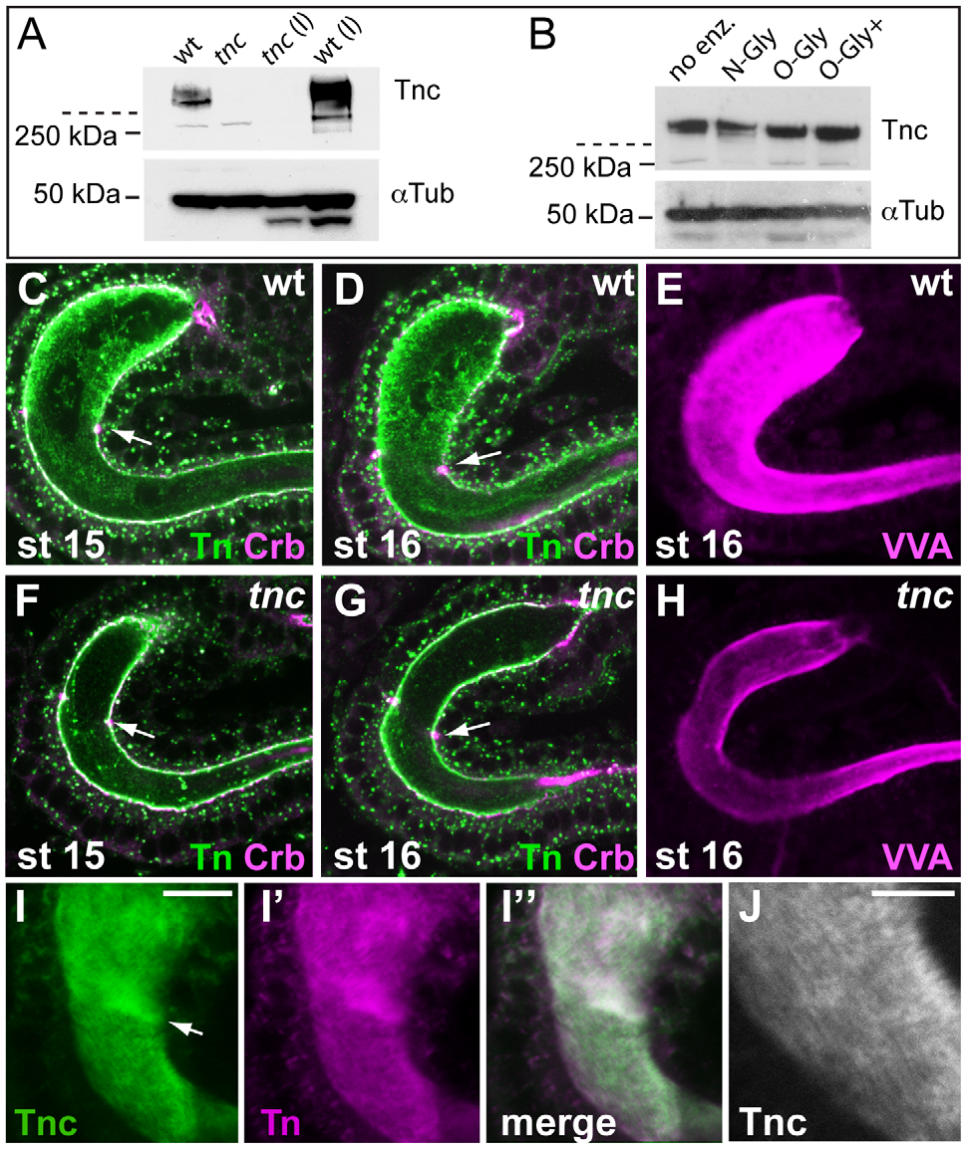
Tnc is a glycosylated intralumial matrix-component. (A) Tnc from wild type and *tnc^13c^* mutant embryos and larvae (l) were detected on western blot and resided as high-molecular weight species in the stacking gel (stippled line indicates end of stacking gel). Anti-α-Tubulin was used as loading control. (B) Protein extracts from stage 16 wild type embryos were subjected to deglycosylation (no enz = no enzyme, N-Gly = N-Glycanase (PNGase F), O-Gly = O-Glycanase, O-Gly+ = O-Glycanase+Sialidase+β(1-4) Galactosidase+β-N-Acetylglucosaminidase). Addition of O-glycanase caused slightly faster migration of Tnc. (C–H) Embryos were co-labelled for the Tn antigen (green) and Crb (magenta) (C, D, F and G) or with VVA (E and H). Anti-Tn stains the wild type lumen with highest intensity in Si at stages 15 and 16 (C and D). The staining is reduced in mutant embryos (F and G). Arrows point to the Si/Li border. VVA also labels the wild type lumen (E) stronger than the mutant lumen (H). The embryos were processed in parallel and the hindgut was imaged at similar views with identical confocal settings. (I) Wild type embryos were prepared with Clark's fixation and stained for Tnc (I, green) and the Tn antigen (I′, magenta). The merged image (I″) shows partial overlap of the staining. Arrow points to the Si/Li border. (J) High magnification of the hindgut in (I), showing a striated pattern of Tnc-staining. Scale bars: 10 µm in I, 5 µm in J.

The water-binding capacity of glycans can cause proteins with densely appended O-glycans to assume voluminous structures upon secretion. Given that Tnc has two large PTS-domains, we investigated if Tnc might be O-glycosylated. The first step in mucin-type O-glycosylation is the α-linked attachment of terminal N-acetylgalactosamine to Serine or Threonine to generate the so-called Tn-antigen, onto which Galactose can be added to generate the T-antigen. The Tnc protein is predicted to have a molecular mass of 290 kDa. When protein extracts from embryos and larvae were analysed, Tnc was retained in the stacking gel as molecular species substantially larger than the 250-kDa marker (Figure 5A), indicating that Tnc carries posttranslational modifications. To test if the larger size could be due to attached O-glycans, we treated embryonic extracts with deglycosylation enzymes. N-glycanase did not affect the migration of Tnc on the gel, but incubation with O-glycanase caused slightly faster migration of Tnc (Figure 5B), suggesting that Tnc is an O-glycosylated protein.

We next asked if accumulation of Tnc in the hindgut lumen contributes to detectable O-glycans. Using an antibody that detects the Tn antigen in embryos resulted in strong staining of many organ lumens, including that of the hindgut. Counter-labelling for Crb showed that anti-Tn stained both the luminal surface and the intraluminal compartment of the hindgut. The intraluminal staining was prominent at stages 15 and 16 (Figure 5C and 5D). When anti-Tn was applied to *tnc^13c^* mutant embryos, there was a marked reduction in intraluminal Tn-staining, although the apical epithelial surface stained at similar intensity in wild type and mutant embryos (Figure 5F and 5G). No other epithelial organs in *tnc* mutant embryos showed visible reduction in anti-Tn-staining (data not shown). The *Vicia villosa* lectin (VVA) also recognizes the Tn-antigen. Like anti-Tnc, labelling with VVA resulted in reduced staining of the hindgut lumen of *tnc^13c^* mutants, when compared to the wild type (Figure 5E and 5H). The fluorescence staining obtained with a conjugate of soybean agglutinin, which binds both terminal α- and β-linked N-acetylgalactosamine and galactopyranosyl residues, did not differ between wild type and mutant embryos (data not shown). These results argue that Tnc is an important carrier of mucin-type O-glycans in the hindgut lumen.

It has been recognized that formalin fixation fails to preserve the texture of glycan-rich matrices, and alcohol-based fixatives are required to demonstrate these structures in glycocalixes [32]. In our experiments, using formalin fixation, both anti-Tnc and anti-Tn resulted in a punctate staining of the hindgut lumen. The use of Clark's fixative with ethanol and acetic acid, however, resulted in dense staining for Tnc in the hindgut (Figure 5I). Upon close examination Tnc appeared as a striated structure that fills the entire hindgut lumen (Figure 5J). Also anti-Tn produced a dense and slightly striated staining of the hindgut lumen when using Clark's fixative (Figure 5I′). Thus, Tnc appears to form a glycan-rich matrix inside the expanding hindgut lumen.

### Misexpression of *tnc* in epithelial tubes promotes lumen dilation

**Figure 6:**
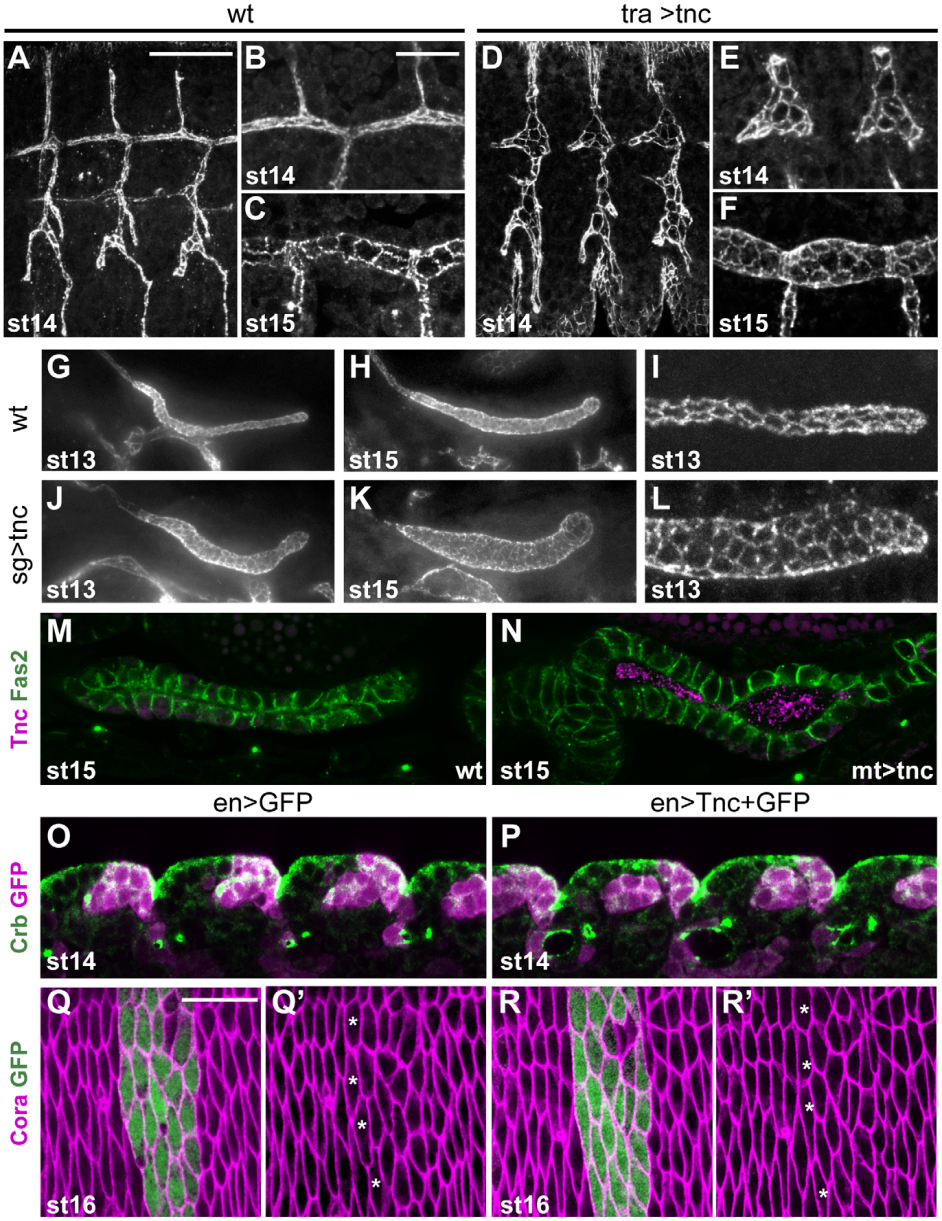
Ectopic expression of *tnc* in tubular epithelia causes excess lumen dilation. (A–F) The apical tracheal surface was visualized by Crb-staining in wild type embryos (A–C) and in embryos with *btlGAL4*-driven *tnc* expression (D–F, tra>tnc). At stage 14, primary branch lumens (A and D) are narrow in the wild type and bloated in tra>tnc embryos, and the dorsal trunk branches (B and E) show altered cell organisation in tra>tnc embryos. At stage 15, tra>tnc embryos have more cells at the lumen perimeter (compare C and F). (G–L) Crb-staining of wild type embryos (G–I) and embryos that express *tnc* in the salivary gland, using *69B-GAL4* (J–L, sg>tnc) shows dilated salivary glands in sg>tnc embryos at stage 13 (G and J) and stage 15 (H and K). The dilation is associated with increased number of cells at the lumen perimeter (I and L). (M and N) *drmGAL4*-driven expression of *tnc* in the malpighian tubule (N, mt>tnc), caused dilated lumens, compared to the wild type (M). The embryos were stained for Fas2 (magenta) that localises to the lateral cell surface and Tnc (green) that is detected in the lumen of mt>tnc embryos. (O–R) *enGAL4* was used to drive expression of GFP (O and Q) or GFP and Tnc (P and R) in epidermal stripes. Double labelling for Crb (green) and GFP (magenta) did not reveal morphological effects of Tnc expression in the epidermis at stage 14 (O and P, lateral epithelial surface). Epidermal cell shape also appears unaffected by Tnc expression, shown by labelling for Cora (magenta) and GFP (green) at stage 16 (Q and R, dorsoventral view, segment 4). The anterior-most cells that express GFP or GFP+Tnc are marked with stars (Q′ and R′) and show similar apical cell circumference as neighbouring non-expressing cells. Scale bars: 20 µm in A (A, D, G, H, J and K), 10 µm in B (B, C, E, F, I, L–P), 10 µm in Q (Q and R). See also Figure S7.

The pan-luminal distribution of Tnc, its dose-dependent function and its ability to cause tube wall expansion, suggested that Tnc might drive tube dilation by causing a luminal pressure. To further investigate this possibility, we asked if misexpression of Tnc in other epithelial tubes, like the trachea, salivary glands and malpighian tubules, would be sufficient to cause tube dilation. The tracheal system arises from invagination of 20 ectodermal cell clusters. During stages 12 and 13, the cells rearrange to build six primary branches without further cell division [33]. At stage 14, branch fusion between neighbouring tracheal metameres form the two dorsal trunks (DTs) and, during stage 15, the trunks expand 3- to 5-fold in diameter [34]. We used *btlGAL4* to drive expression of *UAS-tnc* in tracheal cells from stage 11, which is well before the endogenous onset of tracheal Tnc expression at stage 15. Such tracheal Tnc expression caused excessively dilated primary branches from stage 13 with enlarged apical cell circumferences (Figure 6A, 6B, 6D and 6E). At stage 15, the dorsal trunks in these embryos had more cells at the lumen perimeter than those of the corresponding wild type (Figure 6C and 6F), but the number of tracheal cells was comparable to the wild type situation.

Ectopic expression of *tnc* in salivary glands and malpighian tubules was achieved using *69B-GAL4* and *drmGAL4*, respectively. Each salivary gland arises from a cluster of ectodermal cells that invaginate and form elongated tubes without further cell division [35]. Ectopic expression of *tnc* in the salivary glands resulted in dilated glands, and the dilation became increasingly prominent as development proceeded (Figure 6G, 6H, 6J and 6K). The expansion was accompanied by enlarged apical cell circumferences and an increase in the number of cells encircling the lumen (Figure 6I and 6L). The malpighian tubules, arising from evagination of the hindgut anlage, initially have six to ten cells at the lumen circumference (Janning et al., 1986; Skaer and Arias, 1992) and elongate while the cells rearrange into thin tubes with two cells at the circumference (Skaer, 1993). Expression of *tnc* in the malpighian tubules also resulted in tube dilation (Figure 6M and 6N).

The effects of Tnc on epithelial cell organization in different tubular organs, prompted us to test if Tnc would have a similar influence on epithelial cells in a non-luminal context. We therefore used *69B-GAL4* and *enGAL4* to express *tnc* ubiquitously in the epidermis or in epidermal stripes, respectively. The embryos were stained for Crb and the septate junction protein Coracle (Cora), in order to analyse epidermal morphology and apical cell circumference. The embryos were examined between stages 13 and 16, and we found no anomalies in the appearance of the developing epidermis or in epithelial cell shape of such embryos (Figure 6O–6R, and data not shown), although the embryos expressed Tnc at the apical surface (Figure S7). The ability of Tnc to promote apical surface growth and cell rearrangement when expressed in epithelial tubes, but not in the epidermis, would be consistent with a scenario where Tnc promotes tube dilation by generating an internal luminal pressure.

### Tnc acts non-cell-autonomously and fills the lumen at sites of secretion to cause local tube dilation

**Figure 7:**
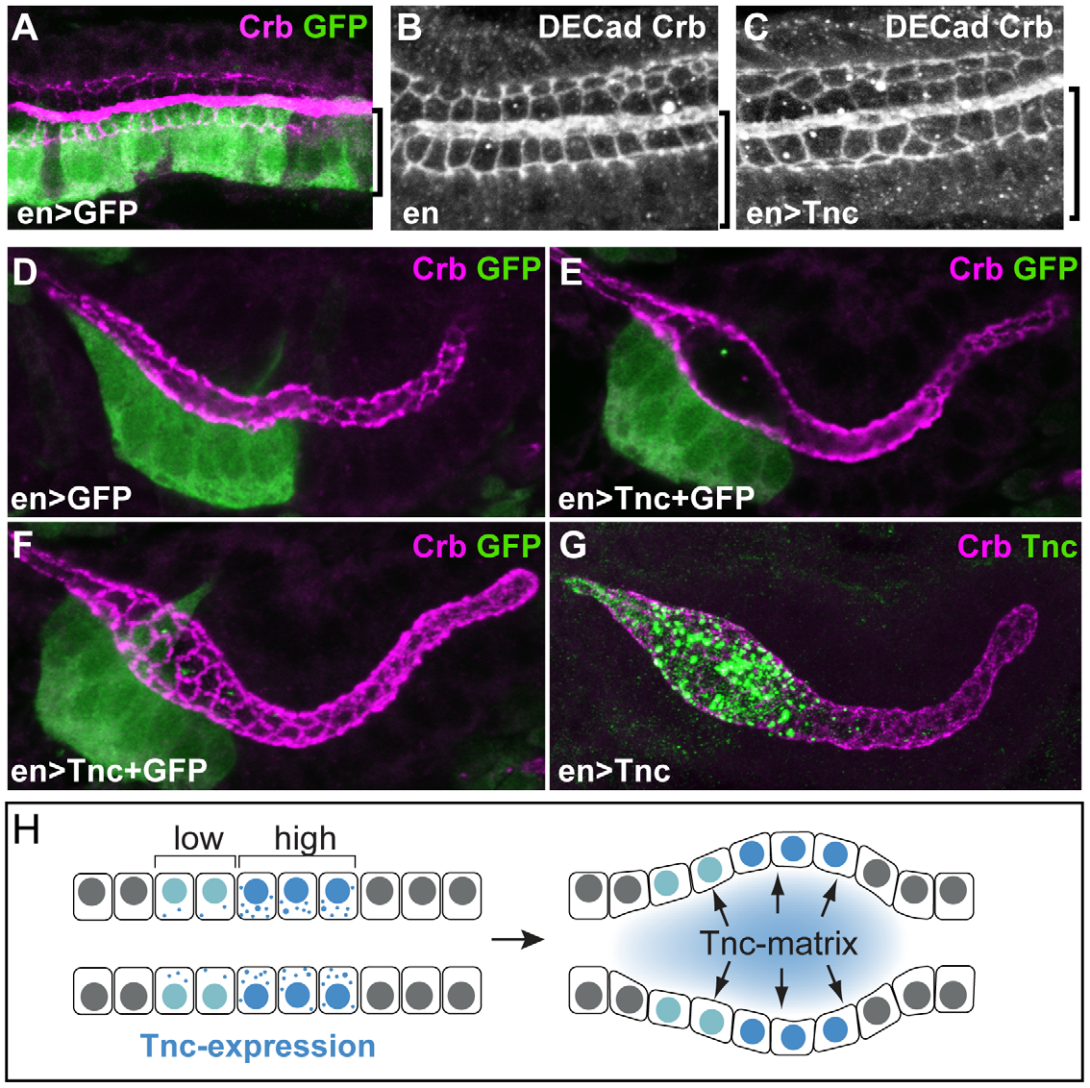
Tnc acts non-cell-autonomously. (A) *enGAL4* drives expression of *UAS-GFP* in the dorsal Li (bracket), as seen by labelling with anti-GFP (green) and anti-Crb (magenta). (B and C) Stage 16 embryos stained for DECad and Crb show that *enGAL4*-driven expression of *tnc* in dorsal Li (bracket) caused enlarged apical cell circumferences in both dorsal and ventral Li (C), when compared to embryos that only express *enGAL4* (B). (D–G) *enGAL4* drives expression of *UAS-GFP* (green) in a cluster of cells in the anterior salivary gland (D, stage 13). *enGAL4*-driven expression of Tnc in salivary glands resulted in local tube dilation (E). By merging serial z-stacked images, the apical cell circumferences were visualized (F). Note that *enGAL4* drives expression in one side of the tube, but all cells at the perimeter show enlarged apical cell circumference. Co-staining for Tnc (green) and Crb (magenta) shows that luminal Tnc localizes to the dilated part of the salivary gland lumen (G). (H) A possible model for the function of Tnc during lumen dilation. Expression of *tnc* in the tubular epithelium causes tube dilation according to the level of expression (“low" and “high") and causes differential dilation along the tube. Once inside the lumen, Tnc acts on surrounding cells, possibly by generating a mechanical pressure, to expand the tube wall.

If Tnc drives tube expansion by adding volume to the lumen, it should promote lumen dilation after its secretion into the lumen and be able to affect cells other than those that produce the protein. In the hindgut, *enGAL4* drives expression selectively in the dorsal Li (Figure 7A). When *enGAL4* was used to over-express *tnc*, the Li lumen diameter became enlarged, while the size of the Si lumen was unaffected (Figure 4F and 4K). Although Tnc was over-expressed only in the dorsal Li, both the ventral and dorsal Li exhibited enlarged apical cell circumferences (Figure 7B and 7C). *enGAL4* also drives expression of UAS-transgenes in a discrete cell cluster at one side of the salivary gland tube (Figure 7D). Expression of *tnc* in this cell cluster, driven by *enGAL4*, resulted in local dilation of the tube (Figure 7E). The cluster of *en*-expressing cells does not span the lumen perimeter but, nevertheless, all cells at the perimeter showed enlarged apical cell circumferences (Figure 7F). Thus, secretion of Tnc by one side of the tube wall promotes cellular changes also in the transverse side of the tube.

Interestingly, when *enGAL4* was used to drive Tnc expression in salivary glands, Tnc was restricted to the dilated part of the tube, where it filled the lumen (Figure 7G). This observation indicates that secreted Tnc forms a local lumen-spanning complex with low mobility, around which dilation occurs. Such behaviour of luminal Tnc would also explain the regional effects of Tnc during normal hindgut tube dilation.

## Discussion

Here, we show that the luminal glycoprotein Tnc promotes diameter expansion of the *Drosophila* hindgut in a dose-dependent manner. The domain organization of Tnc, its contribution to detectable O-glycans in the hindgut lumen and its ability to form a dense luminal matrix suggest that Tnc has mucin-like characteristics. A possible involvement of mucin-like molecules in tubulogenesis has previously been recognized. The *Caenorhabditis elegans* let-653 is a secreted protein with a PTS domain of around 90–200 amino acids, depending on the splice variant. In mutants for *let-653*, the single-celled excretory canals develop massively enlarged lumen by an as yet unknown mechanism [36]. During cyst formation in Madin–Darby Canine Kidney (MDCK), it has been suggested that the initial separation of apical membranes involves de-adhesive properties conferred by large apically localized glycoproteins [37]. Candidate molecules are mucin 1 (MUC1) and the sialomucin Podocalyxin, which localize to the nascent lumens in MDCK cysts and *in vivo* [38], [39]. Recently, it was indeed shown that Podocalyxin is required to separate apical membranes during initial lumen formation in developing blood vessels. Podocalyxin is membrane-bound, and its negatively charged sialic acids are thought to cause electrostatic repulsion of the apical surfaces [17]. Tnc does however appear to function differently from these mucin-like molecules, since it is not required for lumen formation *per se*, but drives the subsequent step of tube diameter expansion.

The function of Tnc also differs from that of the chitinous matrix in the tracheal lumen, as the latter is not needed to increase the luminal volume during diameter expansion, but to shape a uniform diameter [14], [15]. A difference in action between the two luminal components is further supported by the slightly shorter tracheal tubes in *tnc* mutants, while loss of chitin causes too long tracheal tubes. We propose that Tnc-driven tube dilation represents a mechanism for shaping an epithelial tube, where the extent of tube wall extension and lumen volume expansion can be controlled by the intraluminal accumulation of a single protein.

During wing development, Tnc is found basal to the epithelium and is proposed to act as a ligand for PS2 integrin via RGD motifs in the vWC-like domains [23]. It is therefore possibly that luminal Tnc might cause tube wall remodelling by signalling through an apical cognate receptor(s). However, the results do not indicate a signalling function for Tnc: First, over-expression of Tnc in the hindgut causes an increase in tube diameter according to the levels of Tnc expression. Thus, a signaling function of Tnc would imply that Tnc is the limiting factor in the pathway. This is unlikely, since Tnc is abundant and fills the lumen of the wild type hindgut. Second, when Tnc was expressed at one side of the tube wall, all cells at the lumen perimeter were similarly affected. If Tnc signals via an apical receptor, the effects should be higher at the site of its secretion, given its strictly dose-dependent function. Third, the observed lumen-dependent function of Tnc implies that a putative receptor would have to be present in many epithelia in which Tnc is not normally expressed, but yet not ubiquitously, as Tnc had no effect on the epidermis.

Tnc-driven lumen expansion causes an increase in inner and outer tube diameter, associated with epithelial flattening. It is known that luminal volume expansion upon a hydrostatic pressure causes similar effects, for example during inflation of the zebrafish brain ventricle [4], [10], expansion of the mouse blastocyst [2] and *in vitro* growth of renal cysts [6], [7]. Our results would therefore comply with a mechanism whereby luminal accumulation of Tnc forces an increase in lumen volume and, thereby, expansion of the surrounding tube wall. Since luminal Tnc appears to be a major O-glycan with low mobility in the lumen, an attractive hypothesis is that Tnc forms supra-molecular complexes that cause volume expansion due to hydration of the attached O-glycans. Secretion of Tnc into a confined luminal space would then cause a pressure on the tube wall and lumen dilation. In an attempt to further evaluate if the effect of Tnc requires O-glycosylation of the PTS domains, we have analysed hindgut morphology and the size of Tnc in mutants that lack different glycosyl transferases. However, the results were inconclusive, showing effects on both Tnc levels and secretion (Z.S and A.U. unpublished).

The current study also show that Tnc can steer regional differences in tube diameter expansion along the tube axis, according to its pattern of expression. Such a regional effect of Tnc presumably occurs during normal hindgut development, where the amount of Tnc produced by Si is larger than the amount produced by Li. As a likely consequence, Si undergoes a higher degree of diameter expansion than Li, and it also shows a larger reduction in diameter upon loss of Tnc.

In summary, we have shown that Tnc forms a lumen-spanning complex that drives expansion of the surrounding tube wall. The local and dose-dependent effect of Tnc on tube dilation illustrates that a single protein can model differential lumen diameter along a tube. We suggest a model, were Tnc causes a luminal pressure upon secretion and promotes tube dilation according to its voluminous expansion (Figure 7H). Since the lumen of different epithelial organs have been shown to exhibit dynamic patterns of glycan distribution during development [18], [40], [41], it is possible that glycan-rich luminal components have a broad importance in shaping developing epithelial organs.

## Materials and Methods

### Fly stocks and genetics

The P-element *P{EPgy2}EY16369* contains UAS activating elements and is inserted between the two transcriptional start sites of *tnc*, allowing *tnc* transcription to be activated by GAL4 [23]. The *tnc^13c^* and *tnc^130a^* alleles were generated by imprecise excision of *P{EPgy2}EY16369*. Genomic sequencing revealed that the two transcriptional start sites of *tnc* are deleted in *tnc^13c^*, and that the second start site is deleted in *tnc^130a^*. *tnc^81b^* is a precise excision allele and is homozygous viable. A detailed analysis of the different *tnc* alleles generated by excision of *P{EPgy2}EY16369* will be described elsewhere (LB and HB, unpublished). Ectopic-expression of *tnc* was driven by *drumstick-GAL4 (drmGAL4)*, *69B-GAL4*, *engrailed-GAL4 (enGAL4)* and *enGAL4, UAS-GFP* (all from the Bloomington Stock Centre, Indiana, USA) and *BynGAL4* (Iwaki and Judith A. Lengyel, 2002) (from H. Skaer). The deficiency line *Df(3R)BSC655* that uncovers *tnc* was also obtained from Bloomington Stock Centre.

### Immunohistochemistry

Embryos were fixed with 4% formaldehyde for 20 minutes, dechorionated in methanol and stained according to standard procedures. The primary antibodies were: mouse monoclonal IgM 2A12 (1∶10, Developmental Studies Hybridoma Bank, DSHB), rabbit anti-GFP (1∶500; Molecular Probes, MP), rabbit anti-Tnc (1∶1000) [22], mouse monoclonal IgG1 anti-Crb (1∶10; DSHB), mouse monoclonal IgG2a anti-Fas3 (1∶10; DSHB), mouse monoclonal anti-α-Spectrin (1∶10; DSHB), Rabbit anti-Dg (1∶1000; Deng et al., 2003), rat anti-DECad (1∶20; DSHB), mouse monoclonal IgG anti-Delta (1∶10, DSHB), mouse monoclonal IgG1 anti-Engrailed/Invected (1∶20, DSHB), mouse monoclonal IgM anti-Tn 5F4 (1∶20; [42], mouse monoclonal IgG2b anti-Fas2 (1∶20; DSHB) and guinea pig anti-Cora [1∶2000; 43]. For fluorescent visualisation, secondary antibodies from Molecular Probes or Jackson ImmunoResearch were used at 1∶500. Staining with Texas Red-labelled *Vicia villosa* lectin (20 µg/ml; EY Laboratories, Inc. California), Alexa 488-conjugated soybean agglutinin (20 µg/ml; Molecular probes) and fluorescein-conjugated Chitin-binding Probe (1∶500, New England Biolabs) was performed according to manufacturers recommendations. For Clark's fixation, embryos were immersed in ethanol and acetic acid (1∶3) for 5 min, devitellinized and post-fixated in ethanol and acetic for 30 min. The embryos were washed several times in ethanol, rehydrated and subjected to normal staining. Visualization of the hindgut lumen in living embryos was achieved by injecting a 10-kDa dextran dye conjugated with Alexa 594 (Molecular Probes) into the hemolymph at the anterior end of the embryos. After 30 minutes, the dye had leaked into the hindgut lumen, allowing visualization of lumen size. Confocal imaging was done using a Bio-Rad Radiance 2000 system and a Leica DM5500B microscope was used to obtain wide field fluorescent images.

### Western blotting

Proteins from embryos (50 embryos at stage 16) and larvae (5 third instar larvae) were extracted in RIPA lysis buffer. The samples were loaded on a NuPAGE Novex 4–12% Tris-Bis reducing gradient gel (Invitrogen) and the proteins were blotted onto a PVDF membrane. The blots were stained with antisera against the C-terminus of Tnc (1∶5000) or with mouse anti-α-Tubulin (1∶10000, Sigma) and developed using ECL plus (GE-Amersham). Deglycosylation was performed on protein extracts from 50 stage 16 embryos, using an enzymatic deglycosylation kit for N-linked and simple O-linked glycans (GK80110, GK80115, Prozyme, Hayward, CA) according to manufactures instructions.

### In situ hybridisation

Whole-mount in situ hybridization was performed with digoxigenin-labelled RNA anti-sense probes as previously described [14]. The RNA probe used for detection of *tnc* mRNA is the same as probe B of Mur96B/tnc [21] and corresponds to the second mucin-like domain (PCR-amplified from cDNA with 5′GACAATTCCCGAAATCTCCA and 5′CAGCATCCTGAGGAGACACA). A Nikon eclipse E1000 was used for imaging.

### Morphometric analyses of the hindgut

To measure embryonic hindgut lumen dimensions, embryos were labeled with anti-Crb and anti-DECad. Anti-GFP was used to recognize *tnc^13^* homozygous embryos. The embryos were viewed from the dorsal side and z-stacks that spanned the entire hindgut lumen were obtained. Measurements were performed on two-dimensional projections of the z-stacks, using ImageJ. The area of the large intestine (Li) and the small intestine (Si) was determined by tracing the apical epithelial surface based on DECad-staining. The border-cells, highlighted by Crb-staining, were used to demarcate Li and Si. The length of Li and Si was estimated by tracing the left apical surface for Li, and the centre of the lumen for Si. Lumen diameter was derived from area divided by length. All embryos were measured at stage 16, exactly when the four lobes of the midgut had clearly formed and lied parallel to each other (spanning a time window of maximum 15 minutes, as assessed by live imaging at 25°C). The embryos were mounted in Methyl salicylate, which causes a small reduction in embryo size. Since normalization of the measured values to embryo size did not alter the results, the data are presented as true values. Cell numbers per unit length of Li was determined from confocal projections of sections that spanned the upper half of the hindgut tube of embryos labeled with anti-DECad.

## Supporting Information

Figure S1 Detection of Tnc in embryonic epithelial organs (related to Figure 1). (A) Wild type embryos labelled for Tnc (magenta) reveals the presence of Tnc in the lumen of the proventriculus (A, stage 15) and the salivary duct (B, stage 14), but not in the salivary gland (C, stage 15). The epithelium was stained with anti-α-Spectrin (A, green) or with anti-Fas3 (B and C, green). The image in (A) is a transverse view of the proventriculus with arrow and arrowhead pointing to the lumen of the anterior and posterior chambers, respectively. Arrows in B and C point to the lumen. (D) *tnc* mRNA is detected in cardioblasts at stage 16 (bracket, dorsal view). (E and F) RNA in situ hybridization reveals abundant *tnc* expression (blue) in the trachea, hindgut and anal pad of control embryos (*tnc^13c^/Tm3, GFP*) at stage 15 (E). No transcripts are detected in *tnc^13c^* mutant embryos (F). The *tnc^13c^* allele was balanced over a chromosome that carries a GFP transgene in order to enable identification of *tnc^13c^* homozygote embryos by labelling for GFP (brown) prior to in situ hybridization.
(TIF)

Figure S2 Loss of Tnc causes a narrow hindgut diameter (related to Figure 2). (A–D) A wild type embryo (A and C) and an embryo that carry *tnc^13^c* over *Df(3R)BSC655* (B and D) labelled with anti-Fas3 (A and B) were imaged at stage 16, when the four midgut lobes lie parallel to each other (seen by auto-fluorescence in C and D). Note the narrow Si and Li diameter in *tnc^13c^/Df(3R)BSC655* embryos. (E and F) Stage 16 embryos were labelled for Crb (green) and Tnc (magenta). Homozygotes for *tnc^130a^* lack Tnc-staining in the hindgut and develop a narrow hindgut (F), compared to homozygotes for *tnc^81b^* that is a precise excision allele (E). (G and H) Dorsal view of living wild type (G) and *tnc^13c^* mutant (H) embryos at stage 16, which were injected with a 10 kDa dextran dye to visualize epithelial organ lumens. Li and Si are indicated.
(TIF)

Figure S3 Tnc is required for tracheal dorsal trunk elongation (related to Figure 2). (A and B) Stage 16 wild type (A) and *tnc^13c^* mutant (B) embryos were labelled with the tracheal lumen-specific antibody 2A12. The dorsal trunk (arrow) of the wild type is slightly convoluted, while that of the mutant is relatively straight. (C) Tracheal dorsal trunk lengths were measured in stage 16 embryos, when the four lobes of the midgut have rearranged so that the first lobe abuts the fourth (i.e. 45 minutes after the four midgut lobes lie parallel to each other). ImageJ was used to trace the centre of the dorsal trunk lumen from transverse connective 2 to 9. DT lengths were normalized to embryo length. The trachea of *tnc* mutants appeared normal at stage 15, but the dorsal trunks were approximately 10% shorter than those of the wild type at stage 16. (n = 10, p-value<0.05). Scale bar = 20 µm.
(TIF)

Figure S4 Analysis of hindgut length in *tnc* mutant embryos (related to Figure 2). (A and B) Lumen dimensions of Si and Li were measured in embryos stained for Crb, to mark the border cells, and for DECad to highlight the apical surface. Serial z-stacked images spanning the entire hindgut were obtained at dorsal view and merged to show the outline of the lumen. The hindgut of a wild type (A) and *tnc^13c^* mutant embryo (B) at stage 16 are shown with the anterior and posterior border of Li indicated by white lines. (C) Mean lengths of the lumen of Si and Li are shown for the wild type at stages 14 and 16 and for *tnc* mutant embryos at stage 16 (n>5). Error bars represent standard error of mean. * = P-value<0.05. At stage 16, the Li was slightly, but significantly, longer in *tnc^13c^* mutants than in the wild type.
(TIF)

Figure S5 Activity of different GAL4-driver lines in the hindgut (related to Figure 4). Embryos that carry *UAS-CD8:GFP* together with *69B* (A), *Byn-GAL4* (B) or *Drm-Gal4* (C) were stained with anti-GFP. The embryonic hindguts were imaged at early stage 15 (dorsal view) to assess the pattern and relative levels of GFP-expression. Both *69B* and *Byn-GAL4* drive expression in the entire hindgut epithelium. The embryos were collected and stained in parallel and viewed with identical confocal settings. *Byn-GAL4* drives stronger GFP-expression than *69B* in the hindgut epithelium, and *Drm-Gal4* drives strong expression in Si and in the anterior dorsal Li.
(TIF)

Figure S6 Li length upon over-expression of Tnc in the hindgut (related to Figure 4). Mean Li length is shown for wild type embryos and embryos with GAL4-driven *tnc* expression. Error bars represent standard error of mean (n = 8). No significant difference in Li length was observed. P-value<0.05.
(TIF)

Figure S7 Epidermal Tnc expression in en>Tnc embryos (related to Figure 6). Embryos that carry *en-Gal4* and *UAS-tnc* (en>Tnc) were labelled with anti-Tnc to confirm that Tnc is produced in *en*-expressing stripes in the epidermis. (A and B) Ventral views of stage 15 embryos labelled for Tnc. Tnc is detected as stripes in the epidermis in en>Tnc embryos (A, white brackets), but not in wild type embryos (B). Endogenous Tnc expression in the central nervous system is evident in both embryos. (C and D) Confocal imaging of a stage 14 en>Tnc embryo that was labelled for Tnc (C) and Crb (D) shows Tnc at the epidermal surface.
(TIF)

## References

[1] Lubarsky B, Krasnow MA (2003). Tube morphogenesis: making and shaping biological tubes.. Cell.

[2] Watson AJ, Natale DR, Barcroft LC (2004). Molecular regulation of blastocyst formation.. Anim Reprod Sci.

[3] Bagnat M, Cheung ID, Mostov KE, Stainier DY (2007). Genetic control of single lumen formation in the zebrafish gut.. Nat Cell Biol.

[4] Lowery LA, Sive H (2005). Initial formation of zebrafish brain ventricles occurs independently of circulation and requires the nagie oko and snakehead/atp1a1a.1 gene products.. Development.

[5] Tanner GA, McQuillan PF, Maxwell MR, Keck JK, McAteer JA (1995). An in vitro test of the cell stretch-proliferation hypothesis of renal cyst enlargement.. J Am Soc Nephrol.

[6] Li H, Findlay IA, Sheppard DN (2004). The relationship between cell proliferation, Cl- secretion, and renal cyst growth: a study using CFTR inhibitors.. Kidney Int.

[7] Ferrari A, Veligodskiy A, Berge U, Lucas MS, Kroschewski R (2008). ROCK-mediated contractility, tight junctions and channels contribute to the conversion of a preapical patch into apical surface during isochoric lumen initiation.. J Cell Sci.

[8] Gin E, Tanaka EM, Brusch L (2010). A model for cyst lumen expansion and size regulation via fluid secretion.. J Theor Biol.

[9] Wang H, Ding T, Brown N, Yamamoto Y, Prince LS (2008). Zonula occludens-1 (ZO-1) is involved in morula to blastocyst transformation in the mouse.. Dev Biol.

[10] Zhang J, Piontek J, Wolburg H, Piehl C, Liss M (2010). Establishment of a neuroepithelial barrier by Claudin5a is essential for zebrafish brain ventricular lumen expansion.. Proc Natl Acad Sci U S A.

[11] Moriwaki K, Tsukita S, Furuse M (2007). Tight junctions containing claudin 4 and 6 are essential for blastocyst formation in preimplantation mouse embryos.. Dev Biol.

[12] Tsarouhas V, Senti KA, Jayaram SA, Tiklova K, Hemphala J (2007). Sequential pulses of apical epithelial secretion and endocytosis drive airway maturation in Drosophila.. Dev Cell.

[13] Forster D, Armbruster K, Luschnig S (2010). Sec24-dependent secretion drives cell-autonomous expansion of tracheal tubes in Drosophila.. Curr Biol.

[14] Tonning A, Hemphala J, Tang E, Nannmark U, Samakovlis C (2005). A transient luminal chitinous matrix is required to model epithelial tube diameter in the Drosophila trachea.. Dev Cell.

[15] Devine WP, Lubarsky B, Shaw K, Luschnig S, Messina L (2005). Requirement for chitin biosynthesis in epithelial tube morphogenesis.. Proc Natl Acad Sci U S A.

[16] Husain N, Pellikka M, Hong H, Klimentova T, Choe KM (2006). The agrin/perlecan-related protein eyes shut is essential for epithelial lumen formation in the Drosophila retina.. Dev Cell.

[17] Strilic B, Eglinger J, Krieg M, Zeeb M, Axnick J (2010). Electrostatic cell-surface repulsion initiates lumen formation in developing blood vessels.. Curr Biol.

[18] Tian E, Ten Hagen KG (2007). O-linked glycan expression during Drosophila development.. Glycobiology.

[19] Thornton DJ, Rousseau K, McGuckin MA (2008). Structure and function of the polymeric mucins in airways mucus.. Annu Rev Physiol.

[20] Hattrup CL, Gendler SJ (2008). Structure and function of the cell surface (tethered) mucins.. Annu Rev Physiol.

[21] Syed ZA, Hard T, Uv A, van Dijk-Hard IF (2008). A potential role for Drosophila mucins in development and physiology.. PLoS ONE.

[22] Fraichard S, Bouge AL, Chauvel I, Bouhin H (2006). Tenectin, a novel extracellular matrix protein expressed during Drosophila melanogaster embryonic development.. Gene Expr Patterns.

[23] Fraichard S, Bouge AL, Kendall T, Chauvel I, Bouhin H (2010). Tenectin is a novel alphaPS2betaPS integrin ligand required for wing morphogenesis and male genital looping in Drosophila.. Dev Biol.

[24] Iwaki DD, Johansen KA, Singer JB, Lengyel JA (2001). drumstick, bowl, and lines are required for patterning and cell rearrangement in the Drosophila embryonic hindgut.. Dev Biol.

[25] Campos-Ortega JA, Hartenstein V (1997). The Embryonic Development of Drosophila melanogaster.

[26] Tepass U, Hartenstein V (1994). The development of cellular junctions in the Drosophila embryo.. Dev Biol.

[27] Takashima S, Murakami R (2001). Regulation of pattern formation in the Drosophila hindgut by wg, hh, dpp, and en.. Mech Dev.

[28] Castelli-Gair J, Greig S, Micklem G, Akam M (1994). Dissecting the temporal requirements for homeotic gene function.. Development.

[29] Baylies MK, Martinez Arias A, Bate M (1995). wingless is required for the formation of a subset of muscle founder cells during Drosophila embryogenesis.. Development.

[30] Iwaki DD, Lengyel JA (2002). A Delta-Notch signaling border regulated by Engrailed/Invected repression specifies boundary cells in the Drosophila hindgut.. Mech Dev.

[31] Green RB, Hatini V, Johansen KA, Liu XJ, Lengyel JA (2002). Drumstick is a zinc finger protein that antagonizes Lines to control patterning and morphogenesis of the Drosophila hindgut.. Development.

[32] Matsuo K, Ota H, Akamatsu T, Sugiyama A, Katsuyama T (1997). Histochemistry of the surface mucous gel layer of the human colon.. Gut.

[33] Samakovlis C, Hacohen N, Manning G, Sutherland DC, Guillemin K (1996). Development of the Drosophila tracheal system occurs by a series of morphologically distinct but genetically coupled branching events.. Development.

[34] Beitel GJ, Krasnow MA (2000). Genetic control of epithelial tube size in the Drosophila tracheal system.. Development.

[35] Abrams EW, Vining MS, Andrew DJ (2003). Constructing an organ: the Drosophila salivary gland as a model for tube formation.. Trends Cell Biol.

[36] Jones SJ, Baillie DL (1995). Characterization of the let-653 gene in Caenorhabditis elegans.. Mol Gen Genet.

[37] O'Brien LE, Zegers MM, Mostov KE (2002). Opinion: Building epithelial architecture: insights from three-dimensional culture models.. Nat Rev Mol Cell Biol.

[38] Schluter MA, Margolis B (2009). Apical lumen formation in renal epithelia.. J Am Soc Nephrol.

[39] Datta A, Bryant DM, Mostov KE (2011). Molecular regulation of lumen morphogenesis.. Curr Biol.

[40] Minuth WW, Rudolph U (1990). Successive lectin-binding changes within the collecting duct during post-natal development of the rabbit kidney.. Pediatr Nephrol.

[41] Gheri G, Sgambati E, Bryk SG (2000). Glycoconjugate sugar residues in the chick embryo developing lung: a lectin histochemical study.. J Morphol.

[42] Thurnher M, Clausen H, Sharon N, Berger EG (1993). Use of O-glycosylation-defective human lymphoid cell lines and flow cytometry to delineate the specificity of Moluccella laevis lectin and monoclonal antibody 5F4 for the Tn antigen (GalNAc alpha 1-O-Ser/Thr).. Immunol Lett.

[43] Fehon RG, Dawson IA, Artavanis-Tsakonas S (1994). A *Drosophila* homologue of membrane-skeleton protein 4.1 is associated with septate junctions and is encoded by the *coracle* gene.. Development.

