# Supplementary figures and images for: A Luminal Glycoprotein Drives Dose-Dependent Diameter Expansion of the Drosophila melanogaster Hindgut Tube
Source: PLoS Genet. 2012 Aug 2;8(8):e1002850. doi: 10.1371/journal.pgen.1002850 (PMC3410870; doi:10.1371/journal.pgen.1002850)

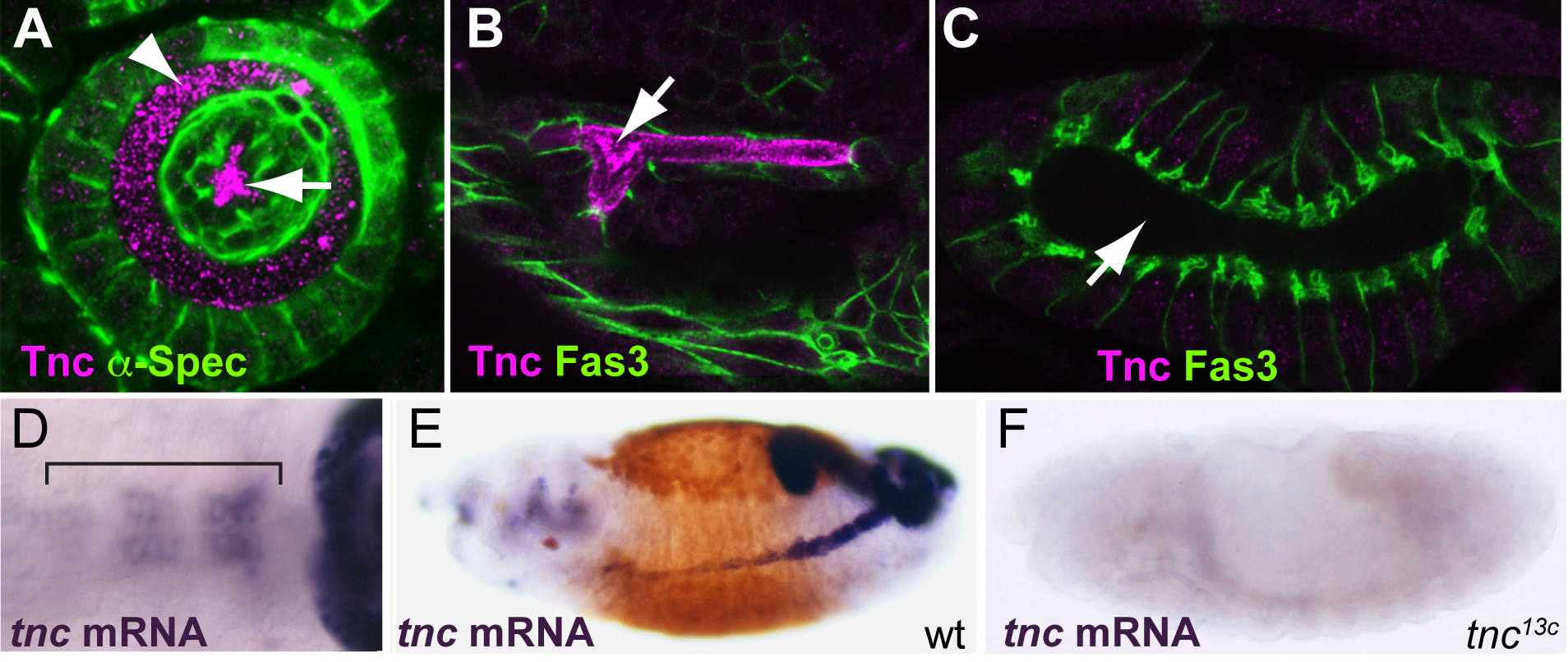

Supplement: Figure S1 — Detection of Tnc in embryonic epithelial organs (related to Figure 1). (A) Wild type embryos labelled for Tnc (magenta) reveals the presence of Tnc in the lumen of the proventriculus (A, stage 15) and the salivary duct (B, stage 14), but not in the salivary gland (C, stage 15). The epithelium was stained with anti-α-Spectrin (A, green) or with anti-Fas3 (B and C, green). The image in (A) is a transverse view of the proventriculus with arrow and arrowhead pointing to the lumen of the anterior and posterior chambers, respectively. Arrows in B and C point to the lumen. (D) tnc mRNA is detected in cardioblasts at stage 16 (bracket, dorsal view). (E and F) RNA in situ hybridization reveals abundant tnc expression (blue) in the trachea, hindgut and anal pad of control embryos (tnc13c/Tm3, GFP) at stage 15 (E). No transcripts are detected in tnc13c mutant embryos (F). The tnc13c allele was balanced over a chromosome that carries a GFP transgene in order to enable identification of tnc13c homozygote embryos by labelling for GFP (brown) prior to in situ hybridization. (TIF) [file pgen.1002850.s001.tif]

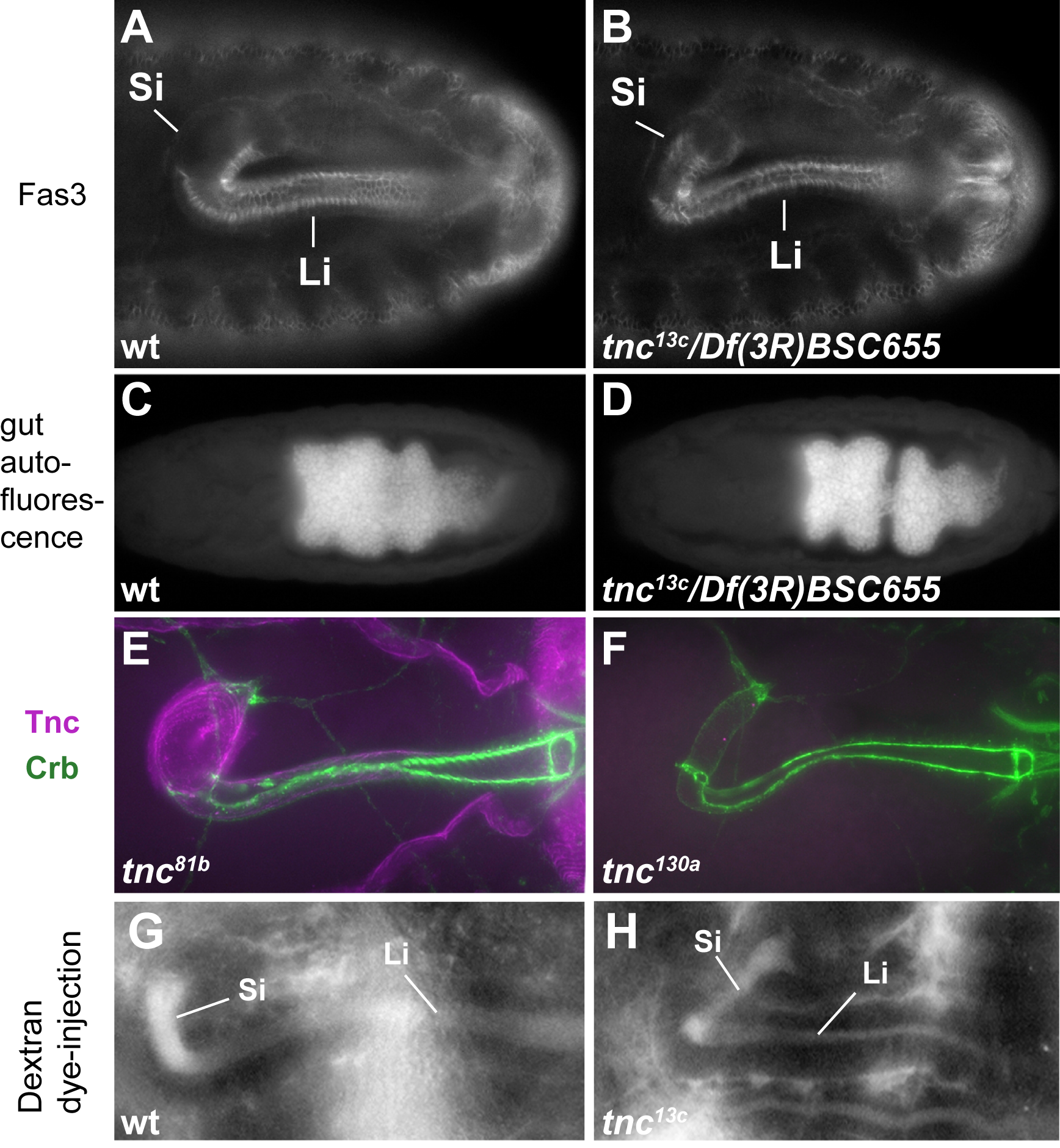

Supplement: Figure S2 — Loss of Tnc causes a narrow hindgut diameter (related to Figure 2). (A–D) A wild type embryo (A and C) and an embryo that carry tnc13c over Df(3R)BSC655 (B and D) labelled with anti-Fas3 (A and B) were imaged at stage 16, when the four midgut lobes lie parallel to each other (seen by auto-fluorescence in C and D). Note the narrow Si and Li diameter in tnc13c/Df(3R)BSC655 embryos. (E and F) Stage 16 embryos were labelled for Crb (green) and Tnc (magenta). Homozygotes for tnc130a lack Tnc-staining in the hindgut and develop a narrow hindgut (F), compared to homozygotes for tnc81b that is a precise excision allele (E). (G and H) Dorsal view of living wild type (G) and tnc13c mutant (H) embryos at stage 16, which were injected with a 10 kDa dextran dye to visualize epithelial organ lumens. Li and Si are indicated. (TIF) [file pgen.1002850.s002.tif]

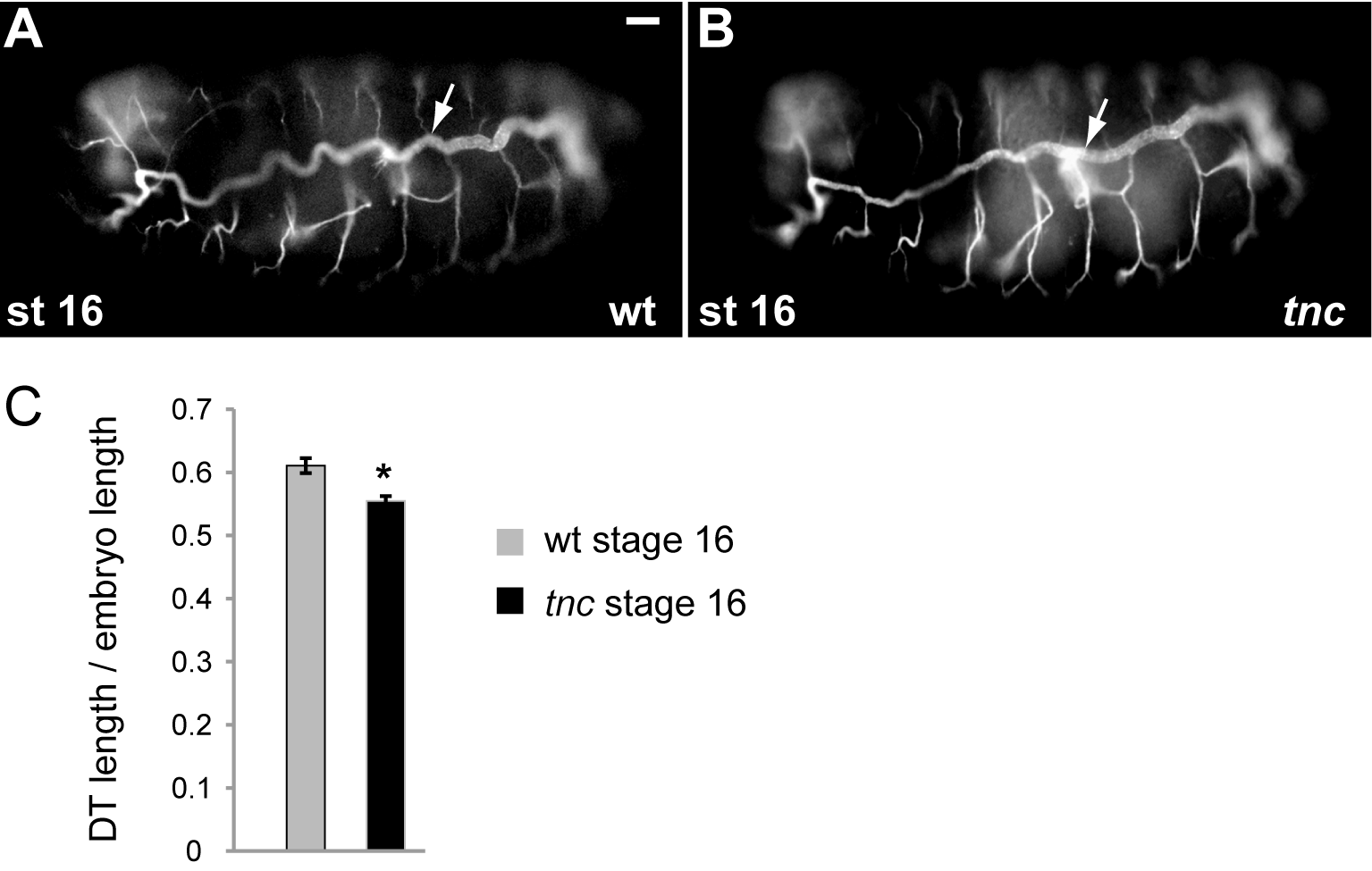

Supplement: Figure S3 — Tnc is required for tracheal dorsal trunk elongation (related to Figure 2). (A and B) Stage 16 wild type (A) and tnc13c mutant (B) embryos were labelled with the tracheal lumen-specific antibody 2A12. The dorsal trunk (arrow) of the wild type is slightly convoluted, while that of the mutant is relatively straight. (C) Tracheal dorsal trunk lengths were measured in stage 16 embryos, when the four lobes of the midgut have rearranged so that the first lobe abuts the fourth (i.e. 45 minutes after the four midgut lobes lie parallel to each other). ImageJ was used to trace the centre of the dorsal trunk lumen from transverse connective 2 to 9. DT lengths were normalized to embryo length. The trachea of tnc mutants appeared normal at stage 15, but the dorsal trunks were approximately 10% shorter than those of the wild type at stage 16. (n = 10, p-value<0.05). Scale bar = 20 µm. (TIF) [file pgen.1002850.s003.tif]

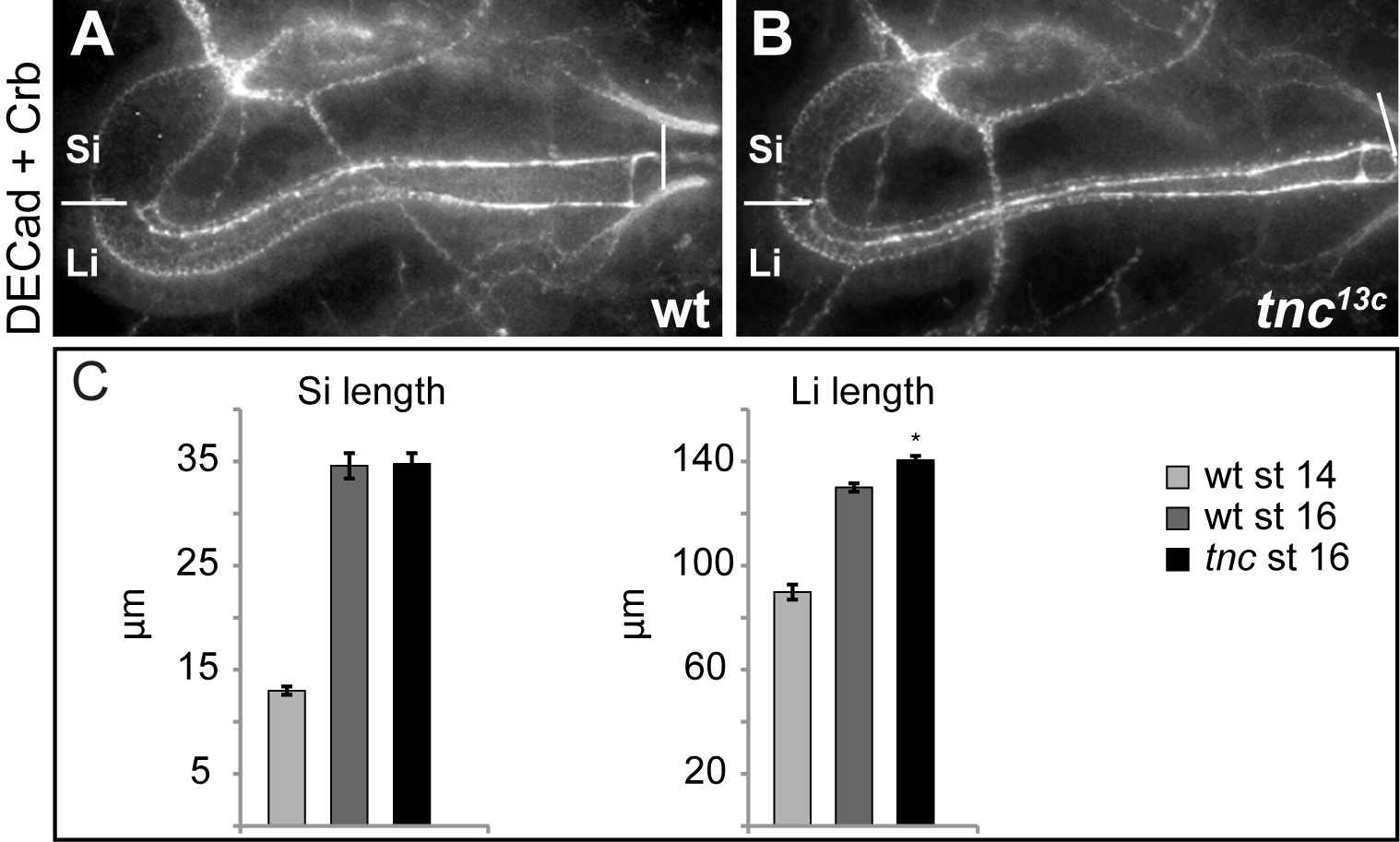

Supplement: Figure S4 — Analysis of hindgut length in tnc mutant embryos (related to Figure 2). (A and B) Lumen dimensions of Si and Li were measured in embryos stained for Crb, to mark the border cells, and for DECad to highlight the apical surface. Serial z-stacked images spanning the entire hindgut were obtained at dorsal view and merged to show the outline of the lumen. The hindgut of a wild type (A) and tnc13c mutant embryo (B) at stage 16 are shown with the anterior and posterior border of Li indicated by white lines. (C) Mean lengths of the lumen of Si and Li are shown for the wild type at stages 14 and 16 and for tnc mutant embryos at stage 16 (n>5). Error bars represent standard error of mean. * = P-value<0.05. At stage 16, the Li was slightly, but significantly, longer in tnc13c mutants than in the wild type. (TIF) [file pgen.1002850.s004.tif]

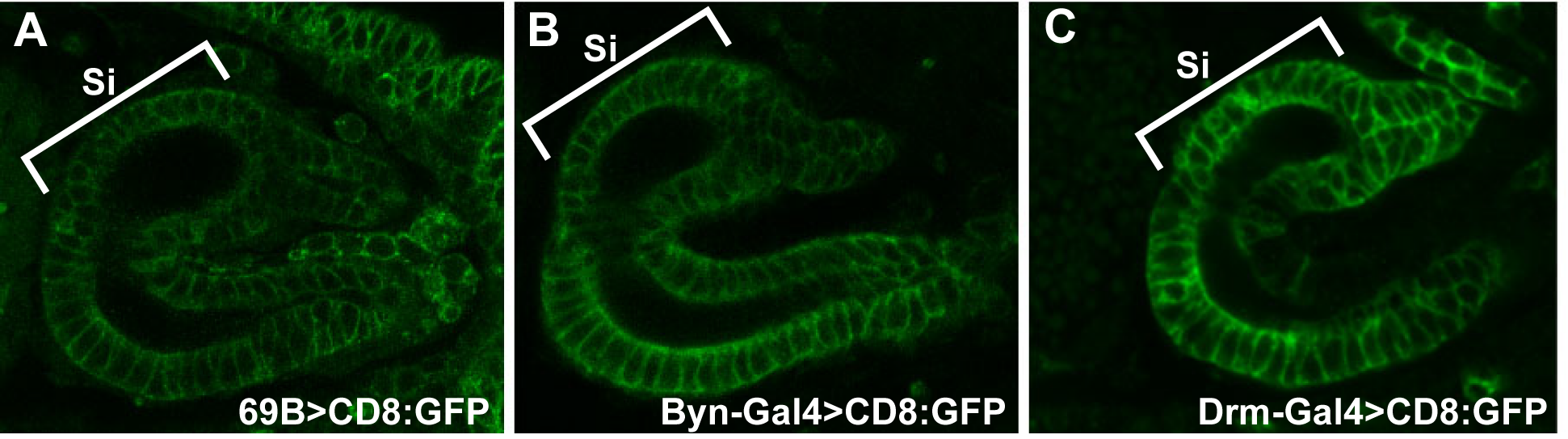

Supplement: Figure S5 — Activity of different GAL4-driver lines in the hindgut (related to Figure 4). Embryos that carry UAS-CD8:GFP together with 69B (A), Byn-GAL4 (B) or Drm-Gal4 (C) were stained with anti-GFP. The embryonic hindguts were imaged at early stage 15 (dorsal view) to assess the pattern and relative levels of GFP-expression. Both 69B and Byn-GAL4 drive expression in the entire hindgut epithelium. The embryos were collected and stained in parallel and viewed with identical confocal settings. Byn-GAL4 drives stronger GFP-expression than 69B in the hindgut epithelium, and Drm-Gal4 drives strong expression in Si and in the anterior dorsal Li. (TIF) [file pgen.1002850.s005.tif]

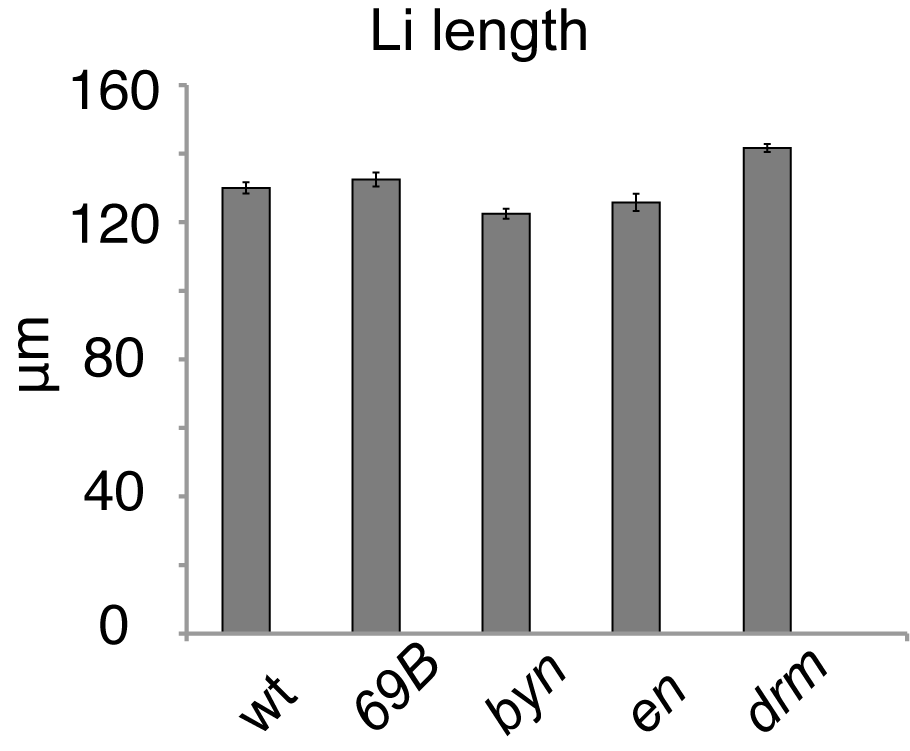

Supplement: Figure S6 — Li length upon over-expression of Tnc in the hindgut (related to Figure 4). Mean Li length is shown for wild type embryos and embryos with GAL4-driven tnc expression. Error bars represent standard error of mean (n = 8). No significant difference in Li length was observed. P-value<0.05. (TIF) [file pgen.1002850.s006.tif]

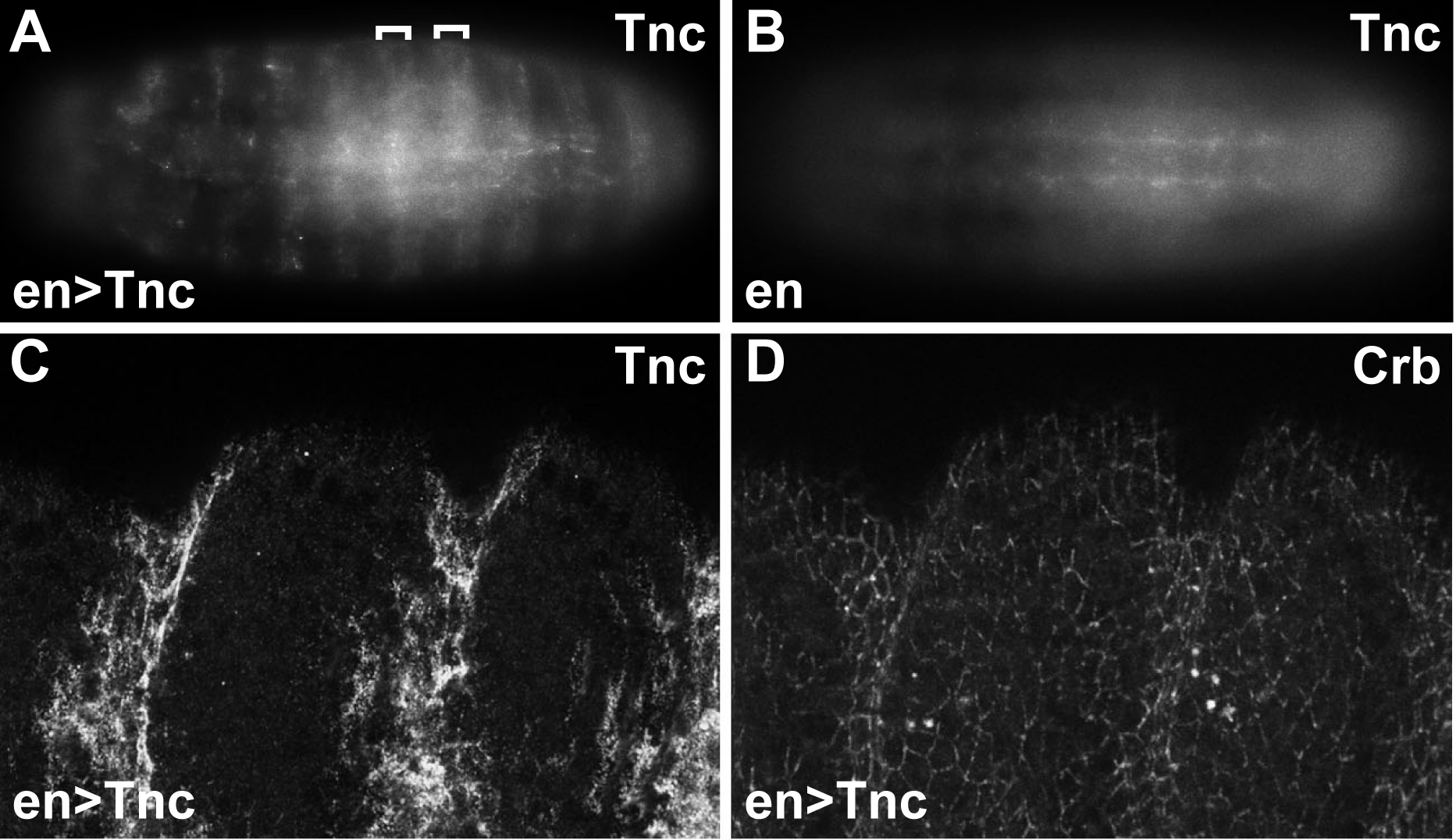

Supplement: Figure S7 — Epidermal Tnc expression in en>Tnc embryos (related to Figure 6). Embryos that carry en-Gal4 and UAS-tnc (en>Tnc) were labelled with anti-Tnc to confirm that Tnc is produced in en-expressing stripes in the epidermis. (A and B) Ventral views of stage 15 embryos labelled for Tnc. Tnc is detected as stripes in the epidermis in en>Tnc embryos (A, white brackets), but not in wild type embryos (B). Endogenous Tnc expression in the central nervous system is evident in both embryos. (C and D) Confocal imaging of a stage 14 en>Tnc embryo that was labelled for Tnc (C) and Crb (D) shows Tnc at the epidermal surface. (TIF) [file pgen.1002850.s007.tif]
